# Joint Modeling of Longitudinal Data and Potentially Informative Visiting Process to Dynamically Predict Future Visit Times

**DOI:** 10.1002/sim.70702

**Published:** 2026-08-09

**Authors:** Achilleas Stamoulopoulos, Christos Thomadakis, Giota Touloumi

**Affiliations:** ^1^ Department of Hygiene, Epidemiology and Medical Statistics Medical School, National and Kapodistrian University of Athens Athens Greece; ^2^ Department of Statistics Athens University of Economics and Business Athens Greece

**Keywords:** dynamic predictions, informative visiting, joint modeling, prediction of future visit times

## Abstract

Frequent visits in cohort studies are important as they enable, among others, the assessment of disease progression and treatment effectiveness. In HIV studies, extended intervals between visits have been shown to be associated with adverse outcomes (e.g., antiretroviral therapy interruption). Prediction of the next visit time can be a useful tool in identifying those more prone to (temporarily) disengage from care. Since visit times are to a large extent individually driven, patient characteristics such as longitudinal markers history and past visit patterns have to be taken into account when dealing with the prediction of future visit times. The topic of dynamic prediction has been developed greatly, especially within the framework of joint models of longitudinal and survival data. While joint models have been extended to account for an informative visiting process, little attention has been given in the dynamic prediction of the next visit time. In this study we propose a model for the joint analysis of a longitudinal continuous marker and the visiting process. A gap time mechanism is employed for the visiting process, while a linear mixed model is used for the marker. The two submodels are linked via correlated normally distributed random effects. The resulting form of the marginal likelihood requires only uni‐dimensional integration, irrespective of the dimension of the random effects in the longitudinal model, a property that holds with multiple continuous markers. The model is used to derive dynamic predictions for the time to the next visit, conditioning on patient's previous gap times and marker history. The performance of the proposed methodology is assessed through simulation. Finally, we apply the proposed model to data of people living with HIV from the Athens Multicenter AIDS Cohort Study.

## 1 Introduction

Patient visits in longitudinal cohort studies are fundamental to enabling the continuous and accurate monitoring of disease progression, particularly for chronic conditions. During these visits, repeated clinical and biomarker measurements are collected, providing a detailed understanding of how a disease evolves and how interventions impact patient health over time. Additionally, frequent visits are essential for the assessment of treatment effectiveness and patient compliance by clinicians, allowing for timely adjustments to optimize patient care.

Our motivating example stems from the HIV infection; in the Athens Multicenter AIDS Cohort Study (AMACS) people with HIV (PWH) receiving antiretroviral therapy (ART) are routinely monitored. During their visits, markers of disease progression, immune function, and inflammation, among others, such as CD4 and CD8 cell counts, HIV‐RNA levels, and serum albumin, are collected. Remaining engaged in care is essential for PWH. Extended intervals between visits, often referred to as gaps in care, are often linked to adverse outcomes, including ART interruption [1]. ART interruption has been shown to be associated with both rapid viral rebound [2] and significantly delayed CD4 restoration after ART reinitiation [3]. AMACS data exploration shows that visit frequency varies significantly between and within individuals, which underscores the challenge of ensuring continuous engagement in care for all individuals. Identifying those at a higher risk of (temporarily) disengaging from care can be a valuable step to prevent such adverse outcomes. Given the substantial variability in visit frequencies, the utilization of patient characteristics, including demographic information, history of longitudinal marker values and previous visit patterns is important for accurately identifying individuals at risk.

Joint models of longitudinal and time‐to‐event data [4] have increasingly being developed and applied for the simultaneous analysis of longitudinal trajectories of one or more (bio)markers and the time to a clinical endpoint, data commonly collected in longitudinal studies. The most widely used subclass of them, shared random effects models, typically consists of two submodels, a longitudinal submodel (e.g., a linear mixed model (LMM)) and a survival submodel (e.g., a log‐normal or a proportional hazards model). Latent random effects or functions of them are used to capture their interrelation. This modeling framework addresses both [5] the bias in longitudinal marker trends which arises when the missingness process is ignored under non‐random dropout scenarios [6], and the endogeneity of these markers, which poses problems when they are used as time‐varying covariates in conventional modeling approaches [7].

Beyond this framework, this class of models has been extended to account for irregular, informative visit times in longitudinal studies. Pullenayegum and Lim [8] proposed a taxonomy for the visiting process, similar to the one proposed by Rubin [9] for missing data mechanisms, according to which visiting is at random (VAR) when visiting at t is independent of marker values at t conditional on data collected up to t, while visiting is not at random (VNAR) when visiting at t depends on marker values at t even when conditioning on data collected up to t. In this context, joint models address VNAR mechanism, relating the longitudinal and the visiting process through shared random effects, aiming to mitigate the bias that may arise in the longitudinal model parameters when the visiting process is ignored. Liang et al. [10] and Sun et al. [11] proposed semiparametric joint models that use nonparametric intercepts in the marker process and correlated random effects in both the visiting and marker processes, with their correlation modeled parametrically in the former, while left unrestricted in the latter. Liu et al. [12] and Zhu et al. [13] considered fully parametric joint models, using shared random effects to link the two processes. The models of Sun et al. and Liu et al. additionally accommodated a terminal event which could depend on both visiting and longitudinal process. All aforementioned models adopted intensity‐based formulations for the visiting process and relied on conditional independence assumptions given the random effects between the marker and the visiting process, implying either VCAR or VNAR mechanism. Recently, Thomadakis et al. [14] proposed parametric joint models in which the visiting process could be modeled by either an intensity‐based or a gap‐time mechanism, which was allowed to depend on both unobserved and observed marker features, thus allowing for all possible visiting mechanisms. In another recent work, Afonso et al. [15] proposed a similar approach jointly modeling longitudinal markers (allowing for non‐Gaussian distributed ones), competing risks, and recurrent clinical events (accommodating both time scales: calendar and gap‐time).

As current emphasis of medical practice towards personalized medicine, a part of the shared random effects joint modeling literature has been focused on the aspect of dynamic, personalized predictions of the time to the clinical endpoint [16, 17]. The estimation of these quantities involves conditioning on the observed patient history, including past longitudinal marker values and baseline characteristics, while they can be dynamically updated as further data are collected. The above characteristics makes them a valuable tool for guiding clinical interventions, empowering physicians to make informed and optimal decisions at each time point; while joint longitudinal and time‐to‐event data models have been used for dynamic prediction of disease progression, the topic of dynamic, personalized prediction of future visit times has received much less attention. In our motivating example such quantities can be particularly useful in identifying individuals at higher risk of prolonged disengagement from care, thus allowing for targeted interventions, and optimizing future visit schedules by selecting time points when patients are most likely to attend. Relative recently, models allowing for the dynamic prediction of recurrent events have been proposed [18, 19]. However, these models, being based primarily on a landmarking framework, do not explicitly account for the relationship between the visiting and the longitudinal process. Thus, the dynamic prediction of future visit times integrating information from previous visit patterns and longitudinal marker history is a unique challenge. The joint modeling framework for longitudinal data and informative visiting processes offers a natural setting for deriving such predictions.

In this article, we propose a joint model for the visiting process and the evolution of a normally distributed longitudinal marker. The model employs a gap time mechanism for the visiting process and can be extended to accommodate multiple longitudinal markers of potentially different types (e.g., binary). Based on the proposed model, we derive personalized predictions for the time‐to‐next visit given all the available information (e.g., for the probability of exceeding a prespecified threshold of clinical importance), including patients' prior visit intervals and longitudinal marker history. The remainder of the paper is organized as follows. In Section 2, we introduce the proposed model, the estimating procedure, extend the model to the case of a binary longitudinal marker and finally demonstrate the estimation of dynamic probabilities for the time‐to‐next visit. In Section 3, we carry out a simulation study to evaluate the performance of the proposed model. In Section 4, the proposed model is applied to post‐ART AMACS data. Finally, in Section 5, we discuss the concluding remarks, limitations and potential extensions of our approach.

## 2 Proposed Model

### 2.1 Longitudinal Marker Model

We consider a continuous longitudinal marker Y. In our context Y can be, for example, the (transformed) CD4 cell counts. To model the evolution of the marker values a standard LMM of the form $Y_{i}(t)=X_{i}^{⊤}(t)\beta +Z_{i}^{⊤}(t)b_{i}+\epsilon_{i}(t)$ is assumed. $Y_{i}(t)$ denotes the observed marker value of individual i at time t, $X_{i}^{⊤}(t)$, and $Z_{i}^{⊤}(t)$ denote the fixed‐ and the random‐effect design matrices at t, respectively, and β, $b_{i}∼N(0,D_{b})$ are the fixed and the random effects, respectively. Finally, $\epsilon_{i}(t)∼N(0,\sigma_{\epsilon }^{2})$ denotes the within‐individual error. Furthermore, assuming that individual i has $n_{i}$ observed visit times at $0=t_{i1}<t_{i2}<\ldots <t_{in_{i}}$ at which marker Y has been measured, $Y_{i}^{⊤}=(Y_{i1},Y_{i2},\ldots ,Y_{in_{i}})$ denotes the observed marker values and, similarly, $X_{i}$ and $Z_{i}$ denote the observed fixed‐ and random‐effect design matrices, respectively. The vector of the marker model parameters can, therefore, be defined as $\theta_{l}^{⊤}=(\beta^{⊤},\text{vech}{\left(D_{b}\right)}^{⊤},\sigma_{\epsilon }^{2})$, where vech is the “vector‐half” operator which stacks the columns of the lower triangular part of a symmetric matrix.

### 2.2 Visiting Process

We denote as $T_{i}$ the follow‐up time and as $w_{ij}=t_{i(j+1)}-t_{ij},j=1,2,\ldots ,n_{i}-1,w_{in_{i}}=T_{i}-t_{in_{i}}$ the between visits gap times, where $w_{ij},j=1,2,\ldots ,n_{i}-1$ are uncensored times and $w_{in_{i}}$ is a right‐censored time, with $w_{i}^{⊤}=(w_{i1},w_{i2},\ldots ,w_{in_{i}})$ being the vector of observed gap times for the ith individual. We assume that $T_{i}$ conditional on the observed history and baseline covariates, is independent of both the longitudinal marker process and the visiting process. Under this assumption, the final gap time $w_{in_{i}}$ is treated as a non‐informatively right‐censored observation. The model for the visiting process takes the form of a proportional hazards model. Specifically, for the jth gap time we assume

(1) $$\begin{array}{cc} & h_{ij}\{w|{\bar{X}}_{i}(t_{ij}),{\bar{Y}}_{i}(t_{ij}),u_{i};\theta_{v}\}\\ & \;=h_{0}(w)\exp\left[\phi^{⊤}{\bar{X}}_{i}(t_{ij})+\gamma^{⊤}g\{{\bar{Y}}_{i}(t_{ij})\}+u_{i}\right]. \end{array}$$

In the Equation (1) w denotes the time since the most recent visit, ${\bar{X}}_{i}(t_{ij})$ is the vector of values of time‐updates covariates observed up to the most recent visit, ${\bar{Y}}_{i}(t_{ij})$ denotes the history of observed values of marker Y up to $t_{ij}$, with g(·) being any vector valued function. For example, the model can include the observed marker values at the most recent visit, that is, $g\{{\bar{Y}}_{i}(t_{ij})\}=Y_{i}(t_{ij})$. $u_{i}$ is a scalar random effect, assumed to follow $N(0,\sigma_{u}^{2})$. Given $u_{i}$ and covariates the gap times of the same individual are assumed be independent (i.e., $u_{i}$ accounts for the potential dependence of the gap times within the same individual). For the baseline hazard we specify a piecewise constant model, that is, $h_{0}(w)=\zeta^{⊤}I(w)$, where $I(w)={\left\{𝕀(k_{0}\leq w<k_{1}),𝕀(k_{1}\leq w<k_{2}),\ldots ,𝕀(k_{K}\leq w<k_{K+1})\right\}}^{⊤}$. Here, $0<k_{1}<k_{2}<\ldots <k_{K}<\infty$ is a set of K prespecified knots that partitions the gap time scale into K+1 intervals, and $\zeta^{⊤}=(\zeta_{1},\zeta_{2},\ldots ,\zeta_{K+1})$ is the vector of the baseline hazard coefficients, the jth element of which represents the value of the baseline hazard in the interval $[k_{j-1},k_{j}),\text{with}\;k_{0}=0,k_{K+1}=\infty$. Therefore, we can denote the visiting model parameters by $\theta_{v}^{⊤}=(\phi^{⊤},\gamma^{⊤},\zeta^{⊤},\sigma_{u}^{2})$.

We link the two models by allowing for non‐zero correlation between the random effects of the two processes and by including past observed marker values in the visiting model as shown in (1). Specifically, we assume that ${\left(u_{i},b_{i}\right)}^{⊤}∼N(0,D)$, where $D=\left(\begin{array}{ll} \sigma_{u}^{2} & D_{ub}\\ {}* & D_{b} \end{array}\right)$, and $D_{ub}$ is the vector of pairwise covariances between $u_{i}$ and the components of $b_{i}$. The full model parameters can then be denoted by $\theta^{⊤}=(\theta_{l}^{⊤},\theta_{v}^{⊤},D_{ub})$. The proposed model suggests a VNAR mechanism if at least one of the components of $D_{ub}$ is different from zero. In the case of all of them being equal to zero, the visiting mechanism is either at random (VAR) or completely at random (VCAR) depending on whether the coefficient(s) of the past marker values (or functions of them) in the (1) are different from zero.

### 2.3 Likelihood Function and Estimating Procedure

Note that in (1) we assume that conditional on all previously observed marker values and the $u_{i}$, the gap‐times are independent of the marker random effects and unobserved marker values. Thus, the conditional on $b_{i}$ and $u_{i}$ joint likelihood of gap‐times and marker values for individual i can be factorized as $f(w_{i},Y_{i}|u_{i},b_{i})=f(w_{i}|u_{i},Y_{i})f(Y_{i}|b_{i})$, where $f(Y_{i}|b_{i})=\prod_{j}f\{Y_{i}(t_{ij})|b_{i}\},f(w_{i}|u_{i},Y_{i})=\prod_{j}f\{w_{ij}|u_{i},{\bar{Y}}_{i}(t_{ij})\}$ follow directly from the LMM and (1).

For inference on the model's parameters, we rely on the marginal joint likelihood of gap‐times and marker values, which can be derived from the conditional, integrating out the random effects $b_{i}$ and $u_{i}$. Using the assumptions made above, noting that $b_{i}|u_{i}∼N\left(\frac{u_{i}}{\sigma_{u}^{2}}D_{ub}^{⊤},D_{b}-\frac{D_{ub}^{⊤}D_{ub}}{\sigma_{u}^{2}}\right)$, the joint marginal likelihood for individual i can be written as

(2) $$\begin{array}{ll} f(w_{i},Y_{i};\theta ) & =\iint f(w_{i},Y_{i}|u_{i},b_{i};\theta )\\ & \;f(u_{i},b_{i};\text{vech}(D_{b}),D_{ub},\sigma_{u}^{2})\;db_{i}\;du_{i}\\ & =\int f(w_{i}|u_{i},Y_{i};\theta_{v})f(u_{i};\sigma_{u}^{2})\\ & \;\left[\int f(Y_{i}|b_{i};\theta_{l})f\{b_{i}|u_{i};\text{vech}(D_{b}),D_{ub},\sigma_{u}^{2}\}\;db_{i}\right]\;du_{i}\\ & =\int f(w_{i}|u_{i},Y_{i};\theta_{v})f(u_{i};\sigma_{u}^{2})\\ & \;f\{Y_{i}|u_{i};\theta_{l},\text{vech}(D_{b}),D_{ub},\sigma_{u}^{2}\}\;du_{i}, \end{array}$$

where

(3) $$\begin{array}{cc} & f(w_{i}|u_{i},Y_{i};\theta_{v})\\ & \;=\left\{\overset{n_{i}-1}{\underset{j=1}{\prod }}h_{ij}(w_{ij}|u_{i})\right\}\exp\left\{-\overset{n_{i}}{\underset{j=1}{\sum }}\int_{0}^{w_{ij}}h_{ij}(s|u_{i})\;ds\right\} \end{array}$$

and

(4) $$\begin{array}{ll} Y_{i}|u_{i} & ∼N\left(\mu_{iY|u},\sum_{iY|u}\right)\\ \mu_{iY|u} & =X_{i}\beta +\frac{u_{i}}{\sigma_{u}^{2}}Z_{i}D_{ub}^{⊤}\\ \sum_{iY|u} & =\sigma_{\epsilon }^{2}I_{n_{i}}+Z_{i}\left(D_{b}-\frac{D_{ub}^{⊤}D_{ub}}{\sigma_{u}^{2}}\right)Z_{i}^{⊤}. \end{array}$$

The assumed form for the hazard model (Section 2.2) permits an analytical calculation of the integral in Equation (3). Consequently, to compute the likelihood contribution for individual i only the uni‐dimensional integral with respect to $u_{i}$ needs to be approximated, substantially reducing the computational burden. For this purpose, we employ Gauss–Hermite quadrature, although (quasi) Monte‐Carlo integration can be utilized as well. We choose to work under the frequentist paradigm maximizing the sample log‐likelihood to obtain the model parameters. In particular, we applied the BFGS algorithm. At each BFGS iteration the integrand in Equation (2) is re‐centered and re‐scaled based on its mode and its second order derivative prior to applying the Gauss–Hermite rules (adaptive Gauss–Hermite method). Typically BFGS requires calculation of the score vector. To avoid the computational burden of numerical approximations, we derived it analytically (Appendix A of the Supporting Information). The matrix D was parameterized in terms of its Cholesky factor, specifically by log‐transforming the diagonal components of it, to ensure positive definitiveness, and leaving the below‐diagonal components untransformed (Log–Cholesky parameterization). The entire approach described above was coded in R. Standard errors of the maximum likelihood estimates were obtained using the Hessian matrix at convergence. To derive 95% confidence intervals for the variance and correlation parameters of the random‐effect terms of the model, we utilize on a Monte‐Carlo simulation approach. Specifically, we generate 1000 samples from the large‐sample distribution of the maximum likelihood‐estimates; at each draw the log‐Cholesky factor of the matrix D is derived, the desired variance and correlation parameters are then appropriately extracted, and finally the confidence interval for each parameter is constructed using the 2.5th and the 97.5th percentiles of the obtained sample.

Notably, extending the model to the case of multiple (possibly correlated) continuous and normally distributed markers, linked to the visiting model as described in Section 2.2, does not increase the dimension of numerical integration. In particular, we can assume standard LMMs for all markers and consider them to be pairwise independent conditional on their respective (normally distributed) random effects, with their correlation being induced through the variance‐covariance matrix of all random effects (similarly we assume that each random effect can have a non‐zero covariance with $u_{i}$). Then, the joint marginal likelihood for individual i has the same form as in Equation (2), with the term $f(Y_{i}|b_{i};\theta_{l})$ in the second equality of Equation (2) being replaced by the product of the likelihoods of all markers conditional on their random effects. Since each of these is a multivariate normal distribution, the integral still represents a multivariate normal distribution with an updated mean vector and variance‐covariance matrix compared to those in Equation (4).

### 2.4 Extension to Binary Longitudinal Marker

We consider now the case where the longitudinal marker Y is binary, that is, $Y_{i}(t)\in \{0,1\}$. An example of a relevant binary marker in our context is the 𝕀(CD4 / CD8>1), as values of the CD4–CD8 ratio lower than 1 have been shown to be predictive of non‐AIDS‐related morbidity and mortality [20]. We postulate a probit mixed model for its evolution, that is, at time t it is assumed that $Y_{i}(t)|b_{i}∼\text{Bernoulli}[\Phi \{X_{i}^{⊤}(t)\beta +Z_{i}^{⊤}(t)b_{i}\}]$, where Φ(·) denotes the cdf of the standard normal distribution. We reformulate it as a latent variable model, that is we introduce the $Y^{*}$ variable such as $Y_{i}^{*}(t)>0|b_{i}\;\Leftrightarrow \;Y_{i}(t)=1|b_{i}$. The above imply a LMM for $Y^{*}$, and in particular $Y_{i}^{*}(t)=X_{i}^{⊤}(t)\beta +Z_{i}^{⊤}(t)b_{i}+\epsilon_{i}^{*}(t)$, with $\epsilon_{i}^{*}(t)∼N(0,1)$. Using the same form as in Section 2.2 for the visiting model, and under the same assumptions we used to derive the likelihood contribution for the i individual in Section 2.3, the likelihood contribution in this case can be again written as $f(w_{i},Y_{i};\theta )=\int f(w_{i}|u_{i},Y_{i};\theta_{v})f(u_{i};\sigma_{u}^{2})f\{Y_{i}|u_{i};\theta_{l},\text{vech}(D_{b}),D_{ub},\sigma_{u}^{2}\}\;du_{i}$. Here,

(5) $$\begin{array}{ll} & f\{Y_{i}|u_{i};\theta_{l},\text{vech}(D_{b}),D_{ub},\sigma_{u}^{2}\}\\ & \;=\int f(Y_{i}|b_{i};\theta_{l})f\{b_{i}|u_{i};\text{vech}(D_{b}),D_{ub},\sigma_{u}^{2}\}\;db_{i}\\ & \;=\int \left\{\overset{n_{i}}{\underset{j}{\prod }}f(Y_{ij}|b_{i};\theta_{l})\right\}f\{b_{i}|u_{i};\text{vech}(D_{b}),D_{ub},\sigma_{u}^{2}\}\;db_{i}\\ & \;=\int \left[\overset{n_{i}}{\underset{j}{\prod }}\mathbb{P}\{A(Y_{ij}^{*})|b_{i};\theta_{l}\}\right]f\{b_{i}|u_{i};\text{vech}(D_{b}),D_{ub},\sigma_{u}^{2}\}\;db_{i}\\ & \;=\int \left[\int_{ll_{1}}^{ul_{1}}\ldots \int_{ll_{n_{i}}}^{ul_{n_{i}}}f({Y^{*}}_{i}|b_{i};\theta_{l})\;dY_{1}^{*}\ldots dY_{n_{i}}^{*}\right]\\ & \;f\{b_{i}|u_{i};\text{vech}(D_{b}),D_{ub},\sigma_{u}^{2}\}\;db_{i}\\ & \;=\int_{ll_{1}}^{ul_{1}}\ldots \int_{ll_{n_{i}}}^{ul_{n_{i}}}\left[\int f({Y^{*}}_{i}|b_{i};\theta_{l})f\{b_{i}|u_{i};\right.\\ & \;\left.\text{vech}(D_{b}),D_{ub},\sigma_{u}^{2}\}\;db_{i}\right]\;dY_{1}^{*}\ldots dY_{n_{i}}^{*}\\ & \;=\int_{ll_{1}}^{ul_{1}}\ldots \int_{ll_{n_{i}}}^{ul_{n_{i}}}f\{{Y^{*}}_{i}|u_{i};\theta_{l},\text{vech}(D_{b}),D_{ub},\sigma_{u}^{2}\},\\ & \;dY_{1}^{*}\ldots dY_{n_{i}}^{*}, \end{array}$$

where

(6) $$A(Y_{ij}^{*})=\left\{\begin{array}{ll} Y_{ij}^{*}\leq 0 & \text{if}\;Y_{ij}=0\\ Y_{ij}^{*}>0 & \text{if}\;Y_{ij}=1, \end{array}\right.$$

(7) $$ll_{j}=\left\{\begin{array}{ll} -\;\infty & \text{if}\;Y_{ij}=0\\ 0 & \text{if}\;Y_{ij}=1 \end{array},\right.\;ul_{j}=\left\{\begin{array}{ll} 0 & \text{if}\;Y_{ij}=0\\ \infty & \text{if}\;Y_{ij}=1 \end{array},\right.$$

and similarly to Equation (4)

(8) $$\begin{array}{ll} {Y^{*}}_{i}|u_{i} & ∼N\left(\mu_{iY^{*}|u},\sum_{iY^{*}|u}\right)\\ \mu_{iY^{*}|u} & =X_{i}\beta +\frac{u_{i}}{\sigma_{u}^{2}}Z_{i}D_{ub}^{⊤}\\ \sum_{iY^{*}|u} & =I_{n_{i}}+Z_{i}\left(D_{b}-\frac{D_{ub}^{⊤}D_{ub}}{\sigma_{u}^{2}}\right)Z_{i}^{⊤}. \end{array}$$

Transition from the second equality to the third in Equation (5) is justified by the equivalence between the probit and the latent normal variable mixed model, while the one from the fourth to the fifth equality follows from Fubini's Theorem. Note that, the multiple integral in Equation (5) represents a multivariate normal probability, the calculation of which can be computed using existing software implementations (e.g., pmvnorm() function in the mvtnorm package in R) through the methodology described by Genz and Bretz [21].

We should mention here that instead of reformulating the probit mixed model, one can instead directly use the likelihood demonstrated in the second equality of Equation (2). In this case, the term $f(Y_{i}|b_{i};\theta_{l})$ is equal to $\prod_{j=1}^{n_{i}}\Phi {\left\{X_{i}^{⊤}(t_{ij})\beta +Z_{i}^{⊤}(t_{ij})b_{i}\right\}}^{Y_{i}(t_{ij})}\times {\left[1-\Phi \{X_{i}^{⊤}(t_{ij})\beta +Z_{i}^{⊤}(t_{ij})b_{i}\}\right]}^{1-Y_{i}(t_{ij})}$, but the nested integral, with respect to $b_{i}$, does not have now a closed‐form solution. However, when considering simple random effects structures as the case of a single random intercept (a common choice in practical settings due to the limited information carried by binary longitudinal variables) this approach may be preferable in terms of computational efficiency since the total dimension of integration needed to derive the joint marginal likelihood in this case is reduced to two. In each case, the estimation procedure can proceed similarly to that in Section 2.3.

### 2.5 Dynamic Predictions for the Time‐to‐Next Visit

For a specific individual i under study with $n_{i}$ past visit times at $t_{i1}<t_{i2}<\ldots <t_{in_{i}}$, corresponding to $n_{i}-1$ observed between visits gap‐times $w_{i}^{⊤}=(w_{i1},w_{i2},\ldots ,w_{i(n_{i}-1)})$ and at which marker values $Y_{i}^{⊤}=(Y_{i1},Y_{i2},\ldots ,Y_{in_{i}})$ have been observed, our objective is to evaluate the probability that the time‐to‐next visit exceeds a threshold c of relevance. We aim to leverage all available information for the individual, that is both previously observed gap‐times and marker values, along with other covariates, hence we focus on the conditional on past gap‐times and marker values probability that the time‐to‐next visit exceeds c, expressed as $\mathbb{P}(w_{in_{i}}>c|w_{i},Y_{i})$. Let $Y_{i,-n_{i}}^{⊤}=(Y_{i1},Y_{i2},\ldots ,Y_{i(n_{i}-1)})$ denote the vector containing the first $n_{i}-1$ marker values for individual i. Assuming the joint model described in Equations ((2), (3), (4)), and by similar arguments as in the likelihood derivation, suppressing conditioning on covariates, this probability can be written as

(9) $$\begin{array}{ll} & \mathbb{P}(w_{in_{i}}>c|w_{i},Y_{i};\theta )\\ & \;=\iint \mathbb{P}(w_{in_{i}}>c|w_{i},Y_{i},b_{i},u_{i};\theta )f(b_{i},u_{i}|w_{i},Y_{i};\theta ),\;db_{i}\;du_{i}\\ & \;=\iint \mathbb{P}\{w_{in_{i}}>c|{\bar{Y}}_{i}(t_{in_{i}}),u_{i};\theta \}\\ & \;\frac{f(w_{i},Y_{i}|b_{i},u_{i};\theta )f(b_{i},u_{i};\theta )}{f(w_{i},Y_{i};\theta )}\;db_{i}\;du_{i}\\ & \;=\frac{\begin{array}{l} \int \mathbb{P}\{w_{in_{i}}>c|{\bar{Y}}_{i}(t_{in_{i}}),u_{i};\theta \}\\ f(w_{i}|u_{i},Y_{i,-n_{i}};\theta )f(u_{i};\theta )\\ \left\{\int f(b_{i}|u_{i};\theta )f(Y_{i}|b_{i};\theta )\;db_{i}\right\}\;du_{i} \end{array}}{f(w_{i},Y_{i};\theta )}\\ & =\frac{\begin{array}{l} \int \mathbb{P}\{w_{in_{i}}>c|{\bar{Y}}_{i}(t_{in_{i}}),u_{i};\theta \}\\ f(w_{i}|u_{i},Y_{i,-n_{i}};\theta )f(u_{i};\theta )f(Y_{i}|u_{i};\theta )\;du_{i} \end{array}}{f(w_{i},Y_{i};\theta )}, \end{array}$$

where the term $f(w_{i},Y_{i};\theta )$ is the marginal likelihood contribution for the ith individual. After fitting the proposed joint model on a random sample and obtaining the maximum likelihood estimates of θ, ${\hat{\theta }}_{ML}$, we substitute them in (9) in order to estimate the probability of interest. The integrals in the numerator and denominator of (9) are calculated using the integrate() function of R, though Gauss–Hermite quadrature rules or Monte‐Carlo integration can be applied as well. To quantify the uncertainty around the point estimate, a Monte‐Carlo pointwise confidence interval can be constructed by calculating the same probability for a large number of θ values sampled from $N\{{\hat{\theta }}_{ML},\hat{var}({\hat{\theta }}_{ML})\}$. The appropriate percentiles of the resulting distribution can be then used as bounds for the confidence interval.

Note that, the above probability is of direct interest when calculated at times very close to individual's last visit. However, if additional time has passed and the patient has not returned for a visit within the next $c^{⋆}$ units of time, it follows that $w_{in_{i}}>c^{⋆}$. Consequently, the relevant probability in this case becomes $\mathbb{P}(w_{in_{i}}>c|w_{in_{i}}>c^{⋆},w_{i},Y_{i}),c>c^{⋆}$. The formula for calculating this probability can be derived with the same arguments as the one in (9). Finally, if one is interested in the whole distribution of $\left\{w_{in_{i}}|w_{i},Y_{i}\right\}$, simulating values of it can be made, utilizing (9) and using inverse transform sampling.

## 3 Simulation Study

### 3.1 Data Generating Mechanisms and Fitted Models

To evaluate the performance of the proposed model's fitting procedure and to assess the potential biases in the visiting models' parameters and in dynamic predictions of the time‐to‐next visit when ignoring the simultaneous evolution of marker values and the dependence of the two processes, a simulation study was conducted.

Longitudinal marker data were generated by an LMM of the form $Y_{i}(t)=(\beta_{0}+b_{i0})+(\beta_{1}+b_{i1})t+(\beta_{2}+b_{i2})\sqrt{t}+\beta_{3}V_{i1}+\epsilon_{i}(t)$, where $V_{i1}∼\text{Bernoulli}(0.5)$ and $\epsilon_{i}(t)∼N(0,\sigma_{\epsilon }^{2})$. For the visiting process, assuming a maximum study duration of 12 years, we simulated between visits gap‐times from a proportional hazards model of the form $h_{ij}(w|u_{i})=h_{0}(w)\exp\{\phi V_{i2}+\gamma Y_{i}(t_{ij})+u_{i}\},j=1,2,\cdots$, where w is the time since the most recent visit at $t_{ij}$, $Y_{i}(t_{ij})$ is the observed marker value at the most recent visit, $V_{i2}∼\text{Bernoulli}(0.5)$ and $h_{0}(w)$ is a common, piecewise‐constant baseline hazard function with two knots at 5 months and 2 years. Random effects $\left(u_{i},b_{i1},b_{i2},b_{i3}\right)$ were generated from a multivariate normal distribution with mean vector equal to 0 and variance‐covariance matrix D with non‐zero pairwise covariances between $u_{i}$ and $b_{i1},b_{i2},b_{i3}$.

Two main scenarios (Scenarios I and II) were considered, each comprising four sub‐scenarios (ScenarioI‐A through ScenarioI‐D, ScenarioII‐A through ScenarioII‐D). The distinction between Scenario I and Scenario II lies in the frequency of visits: Scenario I was characterized by less frequent visits with a median time‐to‐next visit around 10 months, while Scenario II involved more frequent visits with a median time‐to‐next visit of approximately 6 months. The difference was achieved by varying the baseline hazard rate in the (5months,2years) interval between the two scenarios, set to 5.5 for Scenario I and 30 for Scenario II. Within each scenario, the sub‐scenarios (A–D) differed with respect to the assumed correlation between $u_{i}$ and $b_{i1},b_{i2},b_{i3}$. For the sub‐scenario A, we assume $\rho_{u_{i},b_{0i}}=0.45$, $\rho_{u_{i},b_{1i}}=-0.2$, $\rho_{u_{i},b_{2i}}=0.25$ which we refer to as moderate correlation. For the sub‐scenarios B and C we weaken and strengthen respectively the correlations between the random effects by changing $\rho_{u_{i},b_{0i}}$ to 0.25 and 0.7 respectively, referring to them as low and high correlations. Finally, for the sub‐scenario D, we assume the same correlations for $\rho_{u_{i},b_{0i}}$ and $\rho_{u_{i},b_{2i}}$ as in sub‐scenario A, but change the $\rho_{u_{i},b_{1i}}$ to −0.45. For all these scenarios γ was set to −0.1.

For each Scenario 500 datasets of 501 individuals each were generated. In each dataset, the correctly‐specified, proposed joint model was fitted along with a simpler proportional hazards model for the between visits gap‐time, conditional on the random effect $u_{i}$, which ignores the dependence between two processes. Each model was fitted on a training set of 500 individuals, leaving 1 out of the fitting process. For the one individual excluded from the training procedure, we calculate, at his/her last observed visit, the true conditional probability (i.e., under the true‐data generating distributions, evaluated at the true parameter values) of the time‐to‐next visit exceeding 6, 12, 18, and 24 months, using his/her previous observed visits (the number of which varied per dataset). These probabilities, along with their 95% confidence intervals, were also calculated using (a) the estimated parameters from the correctly‐specified joint model, (b) the estimated parameters from the simple proportional hazards model. The bias in the estimated probabilities from the two models was calculated as the absolute difference between the true and the estimated probabilities; the coverage of the 95% confidence intervals was estimated by verifying whether the true probability lay within the estimated interval. As the number of previous visits among validation individuals varied considerably across datasets, we additionally summarized bias and coverage probabilities after stratifying by visit history. Specifically, the 500 validation individuals were divided into three groups according to the number of previous visits ($k\leq k_{1}$, $k\in (k_{1},k_{2}]$, and $k>k_{2}$, where k denotes the number of previous visits). This allowed comparison of individuals with similar amounts of information avoiding averages over heterogeneous information levels. Because visit frequencies differed between Scenarios I and II, we used $k_{1}=7$ and $k_{2}=12$ for Scenario I (less frequent visits), and $k_{1}=12$ and $k_{2}=18$ for Scenario II (more frequent visits). To evaluate the fitted models' performance, we assessed bias, mean squared error (MSE), Monte‐Carlo standard deviation, average model‐based standard error, and empirical coverage probability.

To assess the robustness of our proposed model's fitting procedure to misspecified dependence between the two processes, an additional simulation scenario was explored, generating data from a different joint model. Specifically, marker and gap‐times data were jointly generated under the models $Y_{i}(t)=(\beta_{0}+b_{i0})+(\beta_{1}+b_{i1})t+(\beta_{2}+b_{i2})\sqrt{t+1}+\beta_{3}V_{i1}+\epsilon_{i}(t)$, and $h_{ij}(w|u_{i})=h_{0}(w)\exp\{\phi V_{i2}+\gamma_{1}Y_{i}(t_{ij})+\gamma_{2}b_{0i}+\gamma_{3}m_{i}^{\prime }(t_{ij}+w)+u_{i}\},j=1,2,\ldots$, respectively, with $V_{i1}$ and $V_{i2}$ as before, $m_{i}(t)=(\beta_{0}+b_{i0})+(\beta_{1}+b_{i1})t+(\beta_{2}+b_{i2})\sqrt{t+1}+\beta_{3}V_{i1}$ denoting the underlying marker value at time t, and $m_{i}^{\prime }(t)$ the rate of change of the underlying trajectory at time t. Under this scenario, $u_{i}$ was independently generated from $\left(b_{i1},b_{i2},b_{i3}\right)$, as dependence was captured through $m_{i}^{\prime }(t)$ and $b_{i0}$, whereas $\left(\gamma_{1},\gamma_{2},\gamma_{3}\right)$ was set to (−0.1, 0.25, 0.1). Following the same procedure as previously described, dynamic prediction probabilities were calculated under this true data‐generating mechanism for a particular individual. We fitted three misspecified models: the proposed joint model and two models that completely ignored the dependence between the two processes, namely a simple proportional hazards model for the between‐visit gap times and a plain linear mixed‐effects model for the longitudinal marker trajectory. The estimated parameters from each model, as well as the dynamic predictions obtained from the joint and proportional hazards models were evaluated similarly to the previous simulation settings.

To investigate the performance of the proposed model in the presence of an informative competing terminal event, additional data were generated under Scenario II‐A (frequent visits, moderate correlation), with terminal event times generated from a proportional hazards model of the form $h_{i}^{D}(t)=\lambda \exp\{am_{i}(t)\},$ where $m_{i}(t)$ the underlying value of the considered marker at time t and a=−0.2. This represents a relative strong association between the marker and the terminal event, with a one‐standard‐deviation increase in the underlying marker value being associated with a hazard ratio of approximately 0.3 over the study period. Two sub‐scenarios were considered, with terminal‐event proportions of approximately 5% and 40%. In each simulated sample, the baseline hazard parameter λ was determined numerically so that $\frac{1}{n}\sum_{i=1}^{n}\mathrm{Pr}(\overset{D}{\underset{i}{T}}\leq 12|b_{i})=\rho^{D},$ where $\rho^{D}\in \{0.05,0.40\}$. The proposed model was then fitted while ignoring the terminal‐event process and treating gap times truncated by the terminal event as right‐censored. Performance was assessed using bias, MSE, Monte Carlo standard deviation, average model‐based standard error, and empirical coverage probability.

Finally, to examine the fitting procedure of the proposed joint model in the case where the longitudinal marker is a binary one, an additional scenario was considered. Specifically, we simulated data from the proposed joint model, with marker values being generated from the probit mixed model described in Section 2.4, assuming a single scalar random‐intercept to account for the within‐individual measurements dependence. The correctly specified joint model was fitted to the simulated data, and, as in previous scenarios, performance was assessed by evaluating bias, MSE, Monte‐Carlo standard deviation, average model‐based standard error and empirical coverage probability. For this scenario, a maximum study duration of 10 years was assumed, whereas the correlation parameter $\rho_{u_{i},b_{0i}}$ was set to −0.5, and γ to −4.

### 3.2 Simulation Results

Table 1 summarizes the findings from both a correctly specified joint model and a misspecified model that ignores the simultaneous modeling of the visiting process and longitudinal marker values. Data were generated under the less‐frequent visits scenario with moderate correlation between the random effects of the two processes (Scenario I‐A). The correctly specified model produced approximately unbiased estimates across all fixed‐effect, variance, and correlation parameters of the two processes, achieving adequate coverage rates (94.0%–95.6%). In contrast, the misspecified model, which excluded the longitudinal marker's contribution to the likelihood, exhibited biased estimates for the visiting model parameters. Specifically, the coefficient of the observed marker value in the visiting process model (True: −0.10, Mean estimate: −0.084) and the variance of the random effect u (True: 3, Mean estimate: 2.765) deviated from their true values, with substantially lower coverage rates (2.6% and 77.4%, respectively). For the personalized survival probabilities, the correctly specified joint model yielded estimates closely aligned with the true values, with confidence intervals successfully capturing the true probabilities for most subjects (96.4%–97.4%). In contrast, the misspecified model resulted in some biases in the estimated probabilities, with confidence intervals containing the true values in only a minority of cases (29.9%–30.1%).

**TABLE 1:** Simulation study results, where the data have been generated by the proposed model under Scenario I‐A.

Parameter		Correctly specified						Ignoring longitudinal model					
	True	Est	Bias	MSE	ASE	MCSD	Cov.	Est	Bias	MSE	ASE	MCSD	Cov.
*Longitudinal*													
Intercept ($\beta_{0}$)	17.50	17.490	−0.010	0.129	0.360	0.359	94.0	—	—	—	—	—	—
t ($\beta_{1}$)	−1.10	−1.099	0.001	0.003	0.052	0.052	94.0	—	—	—	—	—	—
$\sqrt{t}$ ($\beta_{2}$)	6.30	6.300	0.000	0.039	0.199	0.198	94.2	—	—	—	—	—	—
$V_{1}$ ($\beta_{3}$)	1.50	1.509	0.009	0.164	0.395	0.406	94.0	—	—	—	—	—	—
*Visiting process*													
Observed marker value (γ)	−0.10	−0.100	0.000	0.000	0.004	0.004	94.8	−0.084	0.016	0.000	0.004	0.004	2.6
$V_{2}$ (ϕ)	0.50	0.494	−0.006	0.014	0.119	0.116	95.6	0.496	−0.004	0.023	0.152	0.150	95.2
*Variance components*													
$\sigma_{u}^{2}$	3	2.982	−0.018	0.047	0.213	0.217	94.2	2.765	−0.235	0.042	0.199	0.204	77.4
$\rho_{u,b_{0}}$	0.45	0.452	0.002	0.002	0.039	0.040	94.2	—	—	—	—	—	—
$\rho_{u,b_{1}}$	−0.2	0.198	0.002	0.003	0.052	0.052	94.6	—	—	—	—	—	—
$\rho_{u,b_{2}}$	0.25	0.247	−0.003	0.002	0.049	0.050	94.8	—	—	—	—	—	—
*Personalized survival probabilities*													
$\mathrm{Pr}(w_{k}>6\text{months}|w,Y)$	—	—	0.3%	—	—	—	96.8	—	1%	—	—	—	30.1
$\mathrm{Pr}(w_{k}>12\text{months}|w,Y)$	—	—	0.5%	—	—	—	97.4	—	1.9%	—	—	—	29.9
$\mathrm{Pr}(w_{k}>18\text{months}|w,Y)$	—	—	0.5%	—	—	—	96.4	—	2.1%	—	—	—	30.1
$\mathrm{Pr}(w_{k}>24\text{months}|w,Y)$	—	—	0.6%	—	—	—	96.6	—	2.2%	—	—	—	29.9

*Note:* Results from 500 replications with each dataset including 500 individuals. “True” denotes the true parameter values; “Est” the mean of the estimates over the 500 replications; “Bias” the mean bias of estimates; “MSE” the mean squared error; “ASE” the average SE; “MCSD” the empirical Monte Carlo deviation of estimates and “Cov.” the empirical coverage probability (%) of the confidence intervals. For the Personalized survival probabilities (where global true values cannot be defined as they depend on specific patients), “Bias” indicates the mean absolute deviation between the true, patient‐specific probability and the estimated one, while “Cov.” the proportion of simulations in which the estimated confidence interval contained the true, patient‐specific probability. Fitting model (i) is correctly specified (“Correctly specified”) and (ii) ignores the longitudinal model (“Ignoring longitudinal model”).

Increasing the correlation parameter between the random intercept and the random effect in the visiting process model during data generation, while keeping all other parameters unchanged (Scenario I‐C), resulted in more notable biases in the misspecified visiting model. The mean estimates for the visiting model parameters shifted further away from the true values (−0.074, 2.492), and the mean absolute difference between true and estimated personalized survival probabilities ranged from 2.6% to 6.5%. This also resulted in even lower coverage rates (Table 2). The correctly specified joint model successfully recovered the true parameter values under this scenario as well.

**TABLE 2:** Simulation study results, where the data have been generated by the proposed model under Scenario I‐C.

Parameter		Correctly specified						Ignoring longitudinal model					
	True	Est	Bias	MSE	ASE	MCSD	Cov.	Est	Bias	MSE	ASE	MCSD	Cov.
*Longitudinal*													
Intercept ($\beta_{0}$)	17.50	17.509	0.009	0.102	0.317	0.319	95.6	—	—	—	—	—	—
t ($\beta_{1}$)	−1.10	−1.098	0.002	0.003	0.052	0.051	95.8	—	—	—	—	—	—
$\sqrt{t}$ ($\beta_{2}$)	6.30	6.294	−0.006	0.038	0.200	0.195	96.0	—	—	—	—	—	—
$V_{1}$ ($\beta_{3}$)	1.50	1.510	0.010	0.040	0.201	0.201	94.2	—	—	—	—	—	—
*Visiting process*													
Observed marker value (γ)	−0.10	−0.100	0.000	0.000	0.004	0.004	95.6	−0.074	0.026	0.000	0.004	0.005	0
$V_{2}$ (ϕ)	0.50	0.502	0.002	0.003	0.060	0.058	94.8	0.499	−0.001	0.021	0.145	0.146	94.0
*Variance components*													
$\sigma_{u}^{2}$	3	2.984	−0.016	0.049	0.219	0.221	93.8	2.492	−0.508	0.040	0.192	0.204	32.4
$\rho_{u,b_{0}}$	0.70	0.697	−0.003	0.001	0.026	0.025	95.0	—	—	—	—	—	—
$\rho_{u,b_{1}}$	−0.2	0.200	0.000	0.003	0.051	0.051	95.2	—	—	—	—	—	—
$\rho_{u,b_{2}}$	0.25	0.251	0.001	0.002	0.049	0.049	95.8	—	—	—	—	—	—
*Personalized survival probabilities*													
$\mathrm{Pr}(w_{k}>6\text{months}|w,Y)$	—	—	0.4%	—	—	—	94.8	—	2.6%	—	—	—	3.4
$\mathrm{Pr}(w_{k}>12\text{months}|w,Y)$	—	—	0.9%	—	—	—	95.2	—	6%	—	—	—	13.2
$\mathrm{Pr}(w_{k}>18\text{months}|w,Y)$	—	—	1%	—	—	—	94.8	—	6.5%	—	—	—	19.7
$\mathrm{Pr}(w_{k}>24\text{months}|w,Y)$	—	—	1.1%	—	—	—	95.2	—	6.5%	—	—	—	24.8

*Note:* Results from 500 replications with each dataset including 500 individuals. “True” denotes the true parameter values; “Est” the mean of the estimates over the 500 replications; “Bias” the mean bias of estimates; “MSE” the mean squared error; “ASE” the average SE; “MCSD” the empirical Monte Carlo deviation of estimates and “Cov.” the empirical coverage probability (%) of the confidence intervals. For the Personalized survival probabilities (where global true values cannot be defined as they depend on specific patients), “Bias” indicates the mean absolute deviation between the true, patient‐specific probability and the estimated one, while “Cov.” the proportion of simulations in which the estimated confidence interval contained the true, patient‐specific probability. Fitting model (i) is correctly specified (“Correctly specified”) and (ii) ignores the longitudinal model (“Ignoring longitudinal model”).

**FIGURE 1:**
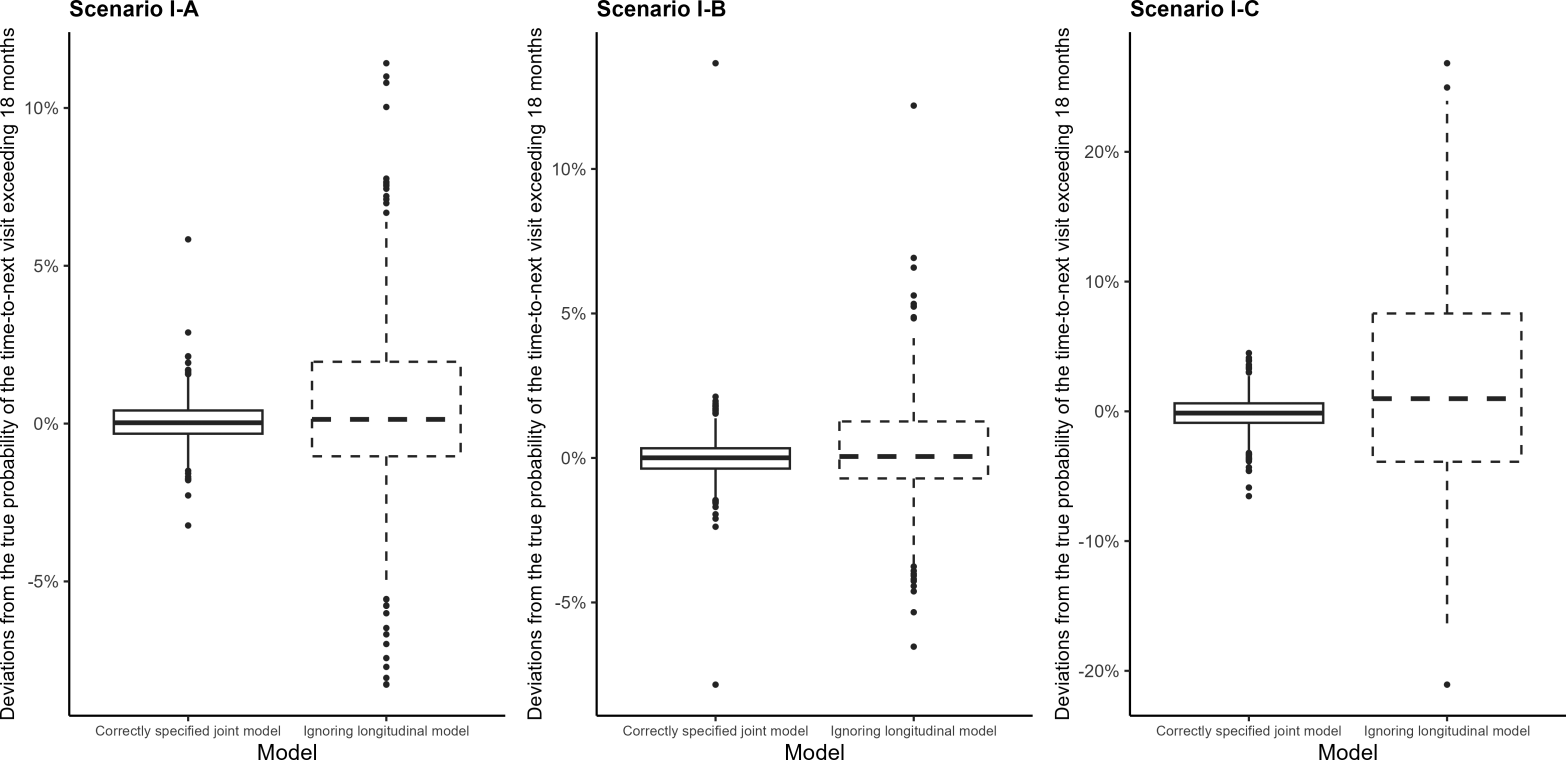
Boxplots for the graphical comparison of the differences between the true dynamic predictions for the time‐to‐next visit exceeding 18 months for the 500 individuals, each of them was left outside the fitting procedure of the respective simulated dataset, and the respective ones estimated from the correctly specified joint model and the one that ignores the longitudinal model, across scenarios I‐A–I‐C.

When a lower correlation parameter between the random intercept and the random effect was assumed during data generation (Scenario I‐B; Appendix B of the Supporting Information), the biases in the misspecified model were naturally less pronounced. The coverage of the last observed marker value coefficient was still low (28%), though. Figure 1 demonstrates the true dynamic prediction of the time‐to‐next visit exceeding 18 months for the 500 individuals, in contrast to the estimates obtained from the correctly specified joint model and the model that considers only the visiting process, under scenarios I‐A–I‐C. Changing the correlation parameter between the random slope and the random effect in the visiting process model from −0.2 to −0.45, while keeping all other parameters consistent with Scenario I‐A (Scenario II‐D; Appendix B of the Supporting Information), still resulted in biases and low coverage rates in the misspecified visiting model, though the extent was reduced compared to Scenario I‐A.

Scenarios involving more frequent patient visits (Scenarios II‐A through II‐D; Appendix B of the Supporting Information) yielded similar conclusions regarding the misspecified visiting model. However, comparing scenarios with identical correlation assumptions but differing visit frequencies (e.g., Scenario I‐A vs. Scenario II‐A) revealed that higher visit frequencies led to less pronounced biases in the misspecified visiting models. This suggests that the additional information provided by more frequent visits can partially alleviate the biases caused by ignoring the joint modeling of longitudinal and visiting data when focusing on making inferences about the visiting process parameters.

When dynamic prediction performance was further evaluated after stratifying individuals by the number of previous visits (Appendix B of the Supporting Information), the correctly specified joint model consistently showed smaller biases and coverage probabilities close to the nominal level across all strata (typically 93%–99%). In contrast, the model ignoring the longitudinal process was poorly calibrated, with coverage frequently below 50% and, in some scenarios involving stronger dependence between the two processes, below 10%. In the less frequent visit scenarios, its performance deteriorated with increasing visit history. For example, in Scenario I‐A, coverage probabilities decreased from approximately 40.8%–42.9% for subjects with fewer previous visits to 7.6%–10.9% for subjects with more visits, while in Scenario I‐C they declined from 7%–8.9% to 0%–1.7%. Conversely, in the more frequent visit scenarios, larger biases were often observed for subjects with fewer previous visits; for example, in Scenario II‐C the mean absolute bias of the estimated probabilities ranged from 2.3% to 10.5% for individuals with fewer previous visits and from 0.2% to 6.5% for those with more extensive visit histories. These findings suggests that the impact of model misspecification depends not only on the strength of the association between the two processes but also on the amount and frequency of the available visit history.

Regarding the data generated under the alternative joint model scenario, both approaches (i.e., our proposed method as well as the simpler ones that ignore the dependence of the two processes) were also fitted (Appendix B of the Supporting Information). We remind that in this scenario the visiting process depended on a different function of the marker's random effects (both the random intercept and the rate of change of the underlying marker trajectory were allowed to affect visiting process model), as well as on the observed marker value at the last visit. Results showed that when the visiting model ignored the longitudinal evolution entirely, the coefficient for the most recent marker value had an average estimate of 0.073, whereas under our proposed misspecified joint model the corresponding mean estimate was 0.104 (true parameter value: −0.10). Biases and coverage rates for the parameters of the visiting process under this scenario are not reported, as linear predictor of the two models were functionally misspecified. With respect to the parameters of the longitudinal model, the fixed‐effect estimates (intercept and time terms) under the simple LMM were biased when the visiting process was ignored (true values: $\left(\beta_{0},\beta_{1},\beta_{2})=(17.5,-1.1,6.3\right)$; mean estimates: (18.3,−0.96,5.66)) with coverage rates of 58%, 43%, and 39.2%, respectively. Such biases were not observed when the proposed joint model was applied; the joint model produced nearly unbiased estimates for the longitudinal submodel parameters, with coverage rates ranging from 95.2% to 96.1%. With respect to the personalized survival probabilities, both the visiting process model which completely ignored the joint evolution of the longitudinal marker, and the proposed joint model exhibited biases (around 2%–3%). Coverage rates were somewhat higher for the proposed joint model (12.4%–41.4% vs. 8.7%–21.4%), but they remained well below acceptable levels.

In the additional simulation study, data were generated under a model including an informative terminal event that was ignored by the proposed joint model. When the terminal‐event incidence was low (approximately 5%), the proposed model produced essentially unbiased estimates for all parameters, with empirical coverage probabilities close to the nominal level (94.0%–97.0%). When the terminal‐event proportion increased to approximately 40%, some departures from the true values became apparent, particularly for the longitudinal submodel. For example, the coefficient of $\sqrt{t}$ showed a bias of 0.171 and coverage decreased to 87.2%. Similar differences were observed for the estimated mean marker level at 12 years, with a bias of 0.392 and coverage of 79.4% for individuals with $V_{1}=1$, and comparable results for $V_{1}=0$. Moderate biases were also observed in some variance and correlation parameters, such as $\sigma_{u}^{2}$ (mean estimate: 2.907 vs. true value: 3.0) and $\rho_{u,b_{2}}$ (mean estimate: 0.226 vs. true value: 0.25), although their coverage probabilities remained reasonably close to the nominal level. In contrast, the visiting‐process parameters were essentially unaffected.

Finally, the estimation procedure demonstrated robustness in recovering the true parameter values when the longitudinal marker was binary (Appendix B of the Supporting Information).

## 4 Application

The proposed model was applied to data of PWH from the Athens Multicenter AIDS Cohort Study. AMACS is a collaborative, open, ongoing, population‐based cohort study, described in detail elsewhere [22]. Specifically, we modeled the post‐ART visiting process of PWH in AMACS, who initiated therapy on or after January 1, 2014, alongside the longitudinal trajectory of CD4 cell counts. The analysis was restricted to individuals aged 18 years or older at ART initiation whose probable HIV exposure route was through either sex between men (MSM) or drug injection (PWID). Transformed, in the square root scale, CD4 counts were modeled through an LMM with a linear (t) and a square root ($\sqrt{t}$) time term in both fixed and random effects to capture their non‐linear evolution, allowing for group‐specific (MSM and PWID) slopes, adjusting further for the age and the calendar year of ART initiation. For the visiting process, the model of Section 2.2 was adopted, using as covariates the observed $\sqrt{\text{CD4}}$ values at the last visit, the probable HIV exposure route, the age at ART initiation, the calendar year of ART initiation and the origin (Greek/Non‐Greek). The baseline hazard was assumed to be piecewise constant with 10 knots at 0.05,0.15,…,0.95 percentiles of the observed gap times. The two models were linked as indicated in Section 2.2. For comparison, two simpler models were fitted; the proportional hazards model for the visiting process considered in the simulation study and the proposed joint model excluding the last observed $\sqrt{\mathrm{CD}4}$ values from the visiting submodel. The models were fitted to 95% of eligible individuals, with the remaining 5% reserved for validation. Model comparison was based on AIC. For the two joint models, we considered ${\text{AIC}}_{VIS|LONG}$, which quantifies the contribution of the visiting process component to the overall AIC while incorporating the additional information provided by the longitudinal data, in the spirit of Zhang et al. [23]. Next, for each validation subject, and using all available patient history known up to the last uncensored gap time, we estimated, under the proposed joint model and the simpler model that ignores the longitudinal marker process, the probability that the subject's final gap time would exceed 9 and 12 months. Knowing the true observed values, these gap times were dichotomized accordingly, and predictive performance was evaluated using the area under the ROC curve (AUC) and prediction error. For this purpose, sensitivity at a given threshold was defined as the proportion of individuals whose predicted probability exceeded the threshold among those whose true gap time exceeded 9 or 12 months, respectively, with specificity defined analogously.

**FIGURE 2:**
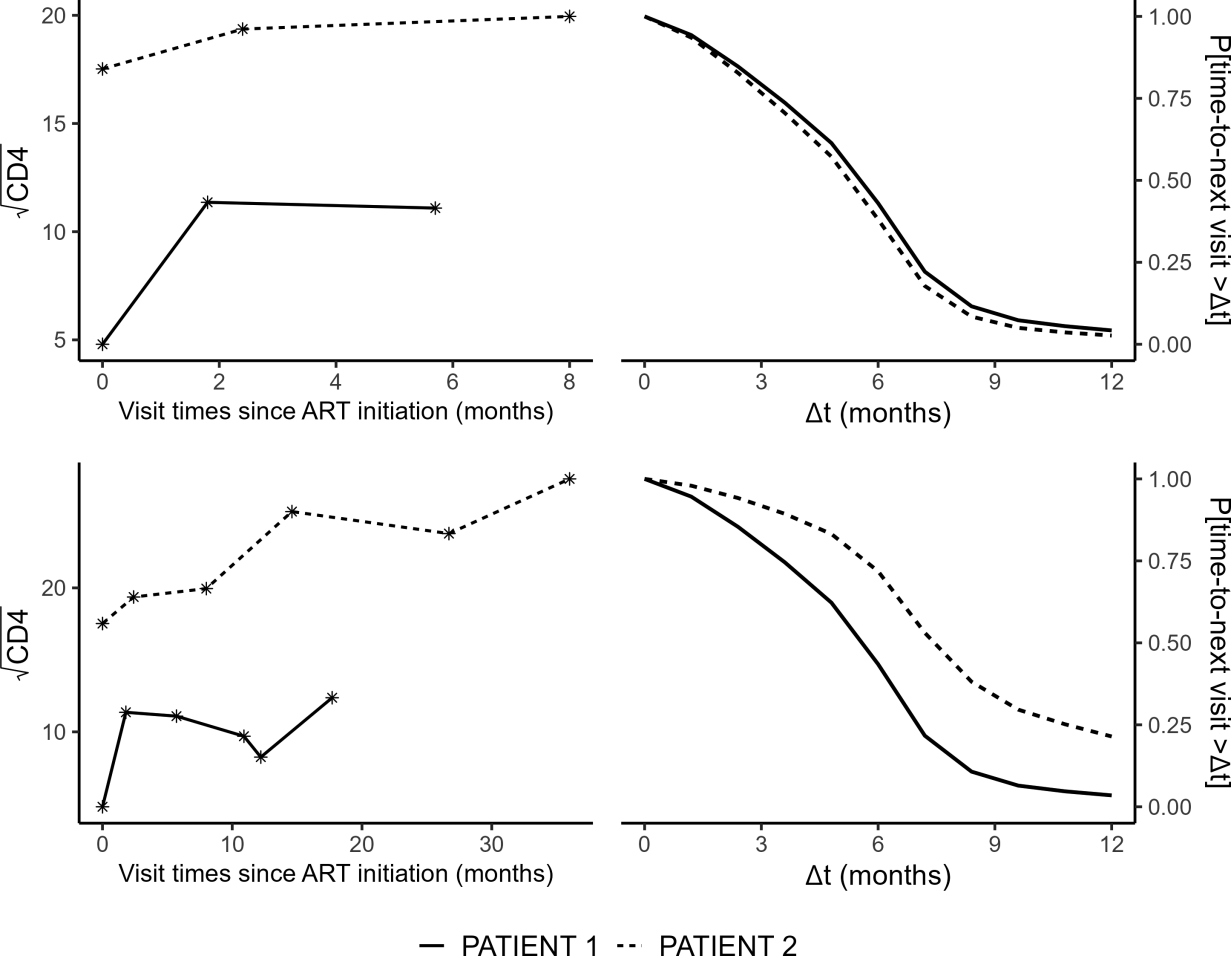
Dynamic prediction for the time‐to‐next visit for PATIENT 1 (PWID of non‐Greek origin) and PATIENT 2 (MSM of Greek origin), estimated at the time points of their follow‐up period where both of them had three (top panel) and six (bottom panel) prior number of visits. Observed CD4 trajectories, up to their last visit, are displayed at the left part of the two panels.

N=2928 individuals were eligible for the analysis, out of whom 83.5% and 16.5% were MSM and PWID respectively. The median (IQR) between visits gap time was 6.5 (2.2) months and 7.5 (5.1) months for MSM and PWID, respectively, while the median (IQR) number of visits was 9 (6, 13) and 6 (4, 11), respectively. Approximately 3% of individuals experienced death during follow‐up. These terminal events were ignored in the present analysis, with the corresponding gap times treated as right‐censored at the time of death. The final gap time was also treated as right‐censored for the 9.3% of individuals who were recorded as lost to follow‐up. The model estimated relatively high correlation between the random effect used in the visiting process and the random intercept, equal to 0.68 (95% CI: 0.65, 0.71), while the two correlation components between the random effect used in the visiting process and the random effect terms of t and $\sqrt{t}$ were low (−0.16 (95% CI: −0.24,−0.08) and 0.16 (95% CI: 0.08, 0.23), respectively). The results suggest that the observed CD4 cell counts have a relatively small yet statistically significant effect on visiting probabilities (Table 3). Specifically, higher observed CD4 counts at the last visit were associated with a decreased hazard of making a visit, with an estimated hazard ratio (HR) per one within‐individual standard deviation of 0.67 (95% CI: 0.66, 0.68). Additionally, MSM were more likely to attend visits more frequently compared to PWID, with the estimated HR for two individuals, one PWID and one MSM, being 0.46, after adjusting for the other factors. Origin was found to be a significant factor for the visiting process as well, with PWH of Greek origin exhibiting a 9% higher hazard of making a visit (95% CI: 1%, 16%) compared to PWH of other origins, controlling for all the other covariates. When considering the simpler model for the visiting process, we noted that the effect of observed CD4 cell counts was underestimated (estimated HR per one within‐individual standard deviation: 0.78, 95% CI: 0.77, 0.79) compared to the corresponding estimate from the joint model. This underestimation aligns with our simulation findings, where ignoring the simultaneous modeling of visiting and longitudinal data led to similar discrepancies to this parameter. The proposed joint model provided the best fit (AIC: 6493.92), outperforming both the model ignoring the longitudinal process (7646.67) and the joint model omitting the observed values from the visiting submodel (9308.78). This suggests that incorporating both the observed marker history and the latent association between the longitudinal and visiting processes improves the description of the visiting mechanism. In the validation sample (Appendix C of the Supporting Information) the joint model appeared to perform marginally better relative to the simpler proportional hazards model. For detecting 9‐month gaps, the joint model achieved an AUC of 0.643 compared with 0.635 for the simpler model. For 12‐month gaps, the joint model again performed better, with an AUC of 0.632 versus 0.605. It should be stressed, however, that this is only an informal comparison of the models. At the time of prediction, each individual has a different number of previously observed gap times. Consequently, the accuracy of model predictions may vary depending on the number of prior events, and this dependence should be reflected in the reported accuracy measures. Finally, Figure 2 illustrates the dynamic predictions for the time‐to‐the next visit for two PWH of the validation set, one MSM of Greek origin and one PWID of non‐Greek origin, calculated at two different time points throughout their follow‐up period at which their number of previous visits was the same. When both patients had three visits (top panel), despite PATIENT 1 being PWID, of no Greek nationality, both factors associated with having lower probability of having a visit, the estimated probabilities of next visit time exceeding time $\Delta_{t}$ was almost identical for the two patients, approaching the value of zero at $\Delta_{t}=$ 12 months. This is explained by the shorter intervals between visits and the lower CD4 counts PATIENT 1 had compared to PATIENT 2. This pattern changed by the sixth visit, where PATIENT 2 exhibited a markedly higher probability that the time to the next visit would exceed $\Delta_{t}$, driven by his higher CD4 trajectory and more sparsely spaced visits (bottom panel), with the probability for $\Delta_{t}=1$ year increasing to approximately 25%.

**TABLE 3:** Results from the proposed joint model fitted to data from the Athens Multicenter AIDS Cohort Study (AMACS).

Parameter	Estimate	95% CI	*p*
*Longitudinal*			
Intercept	23.26	(22.40, 24.14)	<0.001
t (yrs)	−1.01	(−1.10, −0.92)	<0.001
$\sqrt{t}$ ($\sqrt{\text{yrs}}$)	6.38	(6.13, 6.63)	<0.001
Probable HIV exposure route: PWID vs. MSM	−3.14	(−3.83, −2.45)	<0.001
t×Probable HIV exposure route: PWID vs. MSM	0.43	(0.20, 0.67)	<0.001
$\sqrt{t}\times \text{Probable HIV exposure route: PWID vs. MSM}$	−1.76	(−2.40, −1.11)	<0.001
Age at ART initiation (yrs)	−0.12	(−0.14, −0.10)	<0.001
Calendar year of ART initiation: 2017–2019 vs. 2014–2016	1	(0.50, 1.52)	<0.001
Calendar year of ART initiation: ≥2020 vs. 2014–2016	1.67	(1.06, 2.28)	<0.001
*Visiting process*			
Observed $\sqrt{\text{CD4}}$ ($\sqrt{\text{cells}/\mu L}$)	−0.099	(−0.103,−0.096)	<0.001
Probable HIV exposure route: PWID vs. MSM	−0.785	(−0.870,−0.700)	<0.001
Age at ART initiation (yrs)	−0.006	(−0.009,−0.003)	<0.001
Origin: Greek vs. Non‐Greek	0.083	(0.014, 0.152)	0.019
Calendar year of ART initiation: 2017–2019 vs. 2014–2016	−0.054	(−0.123,−0.014)	0.120
Calendar year of ART initiation: ≥ 2020 vs. 2014–2016	0.020	(−0.068, 0.107)	0.654
*Variance & correlation components*			
$\sigma_{u}^{2}$	0.47	(0.43, 0.52)	—
$\sigma_{b_{0}}^{2}$	38.37	(35.99, 40.91)	—
$\sigma_{b_{1}}^{2}$	1.38	(1.17, 1.64)	—
$\sigma_{b_{2}}^{2}$	13.53	(11.70, 15.59)	—
$\rho_{u,b_{0}}$	0.68	(0.65, 0.71)	—
$\rho_{u,b_{1}}$	−0.16	(−0.24,−0.08)	—
$\rho_{u,b_{2}}$	0.16	(0.08, 0.23)	—
$\rho_{b_{0},b_{1}}$	0.02	(−0.06, 0.10)	—
$\rho_{b_{0},b_{2}}$	−0.24	(−0.31, −0.18)	—
$\rho_{b_{1},b_{2}}$	−0.87	(−0.89, −0.84)	—

## 5 Discussion

In this article, we proposed a joint model for the longitudinal evolution of a normally distributed (bio)marker and the visiting process. The visiting model conditions on a subject‐specific random effect, which is assumed to be normally distributed and correlated with the random effects used in the longitudinal model, as well as on past observed marker values. This formulation establishes a straightforward joint likelihood for each individual, requiring only uni‐dimensional integration for computation, regardless of the dimensionality of the random effects in the longitudinal model. This property extends to cases involving multiple normally distributed longitudinal markers. For binary longitudinal markers, we demonstrated that relatively simple likelihood formulations exist. However, for other types of markers, multi‐dimensional integration remains necessary, similar to standard joint modeling approaches. Based on the proposed model, we derived dynamic predictions for the time‐to‐next visit, conditioning on previously observed between‐visits times of the patient, observed marker value history and baseline covariates. We explored the impact of ignoring the simultaneous marker and visiting process modeling on the inference for the visiting model parameters when the true data generation mechanism follows the proposed model. In particular, we identified biases and low coverage rates in the coefficient of the past marker value in the visiting model and the variance of its random‐effect term. These issues were more pronounced when the correlation of the random effects in the longitudinal and the visiting model was stronger, specifically the correlation between the random intercept and the random effect of the visiting model. In scenarios with more frequent patient visits the deviations from the true values and the low coverage rates seemed to be alleviated to some extent. A similar pattern of bias was also observed in the dynamic predictions of the time‐to‐next visit when these were calculated by a model that ignored the simultaneous longitudinal marker evolution. A particular scenario where the proposed model was fitted to data generated under an alternative joint model was also explored. In this setting, our proposed joint model, even misspecified, managed to correctly estimate several key parameters of interest (e.g., longitudinal model parameters) and retain their nominal coverage probabilities, unlike the simpler approaches that ignored the simultaneous modeling of longitudinal and visiting process. However, dynamic predictions still exhibited bias, although to lesser extent compared to those derived from the simpler model of the visiting process. As with any parametric model, the performance of the proposed model depends on the adequacy of its underlying assumptions, and some sensitivity to model misspecification is expected. Nevertheless, the bias in these predictions was not that severe, and generally the coverage of such predictions is not well studied in the literature, especially under misspecification. In any case, it should be mentioned that the calculation of dynamic predictions for the time to the next visit, conditional on the marker and visiting history, can be straightforwardly extended to more complex joint modeling structures with suitable modifications. Regarding computational time, fitting the proposed model required approximately 16 s for a typical simulated dataset under the frequent‐visit scenarios (approximately 8,0009,000 rows; 12‐point Gauss–Hermite quadrature; 20 estimated parameters) and 167 s for the AMACS application (25,682 rows; 12‐point Gauss–Hermite quadrature; 37 estimated parameters). Computations were performed on a computer equipped with a 12th Gen Intelö Core i7‐12700 processor and 32 GB RAM.

It is also worth clarifying the relationship between the present work and that of Thomadakis et al. [14]. Although, both studies jointly model longitudinal markers and potentially informative visiting processes, they differ in their modeling strategy. Thomadakis et al. model the dependence between the longitudinal and visiting processes through latent marker characteristics, previous observed marker values, and functions of previous visits. In contrast, we adopt a correlated shared random‐effects formulation that is computationally attractive as only uni‐dimensional integration is required, and naturally facilitates dynamic prediction of future visit times—the primary objective of the present work—while likewise incorporating previous observed marker values in the visiting submodel. Unlike Thomadakis et al., who account for informative terminal events, we assume terminal events are non‐informative. When this assumption is violated, bias may arise in both the longitudinal [6] and visiting submodels [24]. Our additional simulation study showed that omitting the terminal event led to bias and undercoverage, particularly for longitudinal‐model parameters, when terminal‐event incidence was high, whereas the impact was minimal when incidence was low. Because death was relatively uncommon in AMACS (approximately 3%), we expect this assumption to have little impact in the present application.

Although, the focus of this paper is on the visiting part of the model and on dynamic predictions, we should note that the proposed model falls within the category of shared parameter models used in the literature to overcome possible biases in the longitudinal model under an informative visiting process. As we discussed, the possible correlation between the random effects of the marker and the random effect used in the visiting model suggests a visiting not random mechanism. Although, these correlations may not have an appealing interpretation, the model can be still used, as part of a sensitivity analysis, to examine the visiting process mechanism and possible differences in the longitudinal model parameters. This was illustrated in the different data‐generating scenario, where the misspecified proposed model was nevertheless able to recover the true marker trajectory. Furthermore, even after conditioning on the random effects, the visiting process was not assumed independent of the longitudinal marker. Instead, it was allowed to depend on specific functions of the observed marker, thereby relaxing the conditional independence assumption often imposed in joint modeling, accommodating a VAR mechanism as well. We also note the potential competition for information between the coefficient(s) of the observed marker functions in the visiting submodel and the parameters of $D_{ub}$, which may lead to instability in finite samples. No such instability was observed in either the simulation study or the AMACS application. In the latter, the model including both components provided a better fit than either nested model obtained by omitting one component, while only moderate correlations between the corresponding estimators were observed, suggesting stable estimation. Nevertheless, if instability arises in other finite‐sample settings, the two nested models considered in the application may provide useful complementary analyses.

Regarding possible extensions of the proposed approach, incorporating informative terminal events through additional submodels that depend on characteristics of the marker(s) and/or the visiting process could be a valuable direction for future research. Furthermore, we did not extend the proposed model to accommodate a different time scale for the visiting process model. While gap‐time scale models are useful when the interest lies in the prediction of the next event [25], a calendar time scale could be utilized as well. A possibly interesting aspect is to explore how the choice of an incorrect time scale (i.e., a gap time scale while the underlying time scale is that of calendar time and vice versa) impacts the dynamic predictions of the time‐to‐next visit. We should mention here, though, that the work from Thomadakis et al. [14] suggest, in general, greater robustness of the gap‐time scale in cases where the time scale is misspecified.

From a practical perspective, one of the motivations of this work was to be able to track individuals (PWH in our case) more prone to remain out of care for extended periods. The model was developed under the assumption that visits are not scheduled in advance, but are mainly individually driven. If information on scheduled visits exists the proposed model can still be useful and a possible way to incorporate this information into the current model is as a (time‐updated at each visit) covariate for the visiting model, along with an (also time‐updated) covariate reflecting punctuality on attending previous scheduled visits. In such cases, relevant dynamic predictions include probabilities of the form $\mathbb{P}(w_{in_{i}}>c_{i}|w_{i},Y_{i})$, where now $c_{i}$ represents the individually assigned time till the next visit, or $\mathbb{P}(w_{in_{i}}\in (c_{i}-\epsilon ,c_{i}+\epsilon )|w_{i},Y_{i})$ where ϵ represents an acceptable tolerance window (e.g., a week) around the scheduled visit within which attendance is not considered as being out of care. A valuable mean for better utilizing the dynamic predictions and, ultimately, developing a tool that can accurately identify individuals more prone to remain out of care is to adapt standard accuracy measures, such as sensitivity, specificity, AUC, Brier Score, in the framework described here. Such measures have been adopted generally in the joint modeling framework [26, 27]. In our context, for a given k and threshold $\Delta_{t}$, a case is defined as an individual whose kth gap time exceeds $\Delta_{t}$, whereas a control is an individual whose kth gap time does not. The prediction rule can be based on the probability $\mathbb{P}(w_{k}>\Delta_{t}|w,Y)$. As discussed in the Results section, the associated accuracy measures should depend not only on $\Delta_{t}$ but also on the value of k (reflecting the previous number of visits), to ensure comparability of the individuals contributing to their calculation and allow the model demonstrate varying accuracy depending on the amount of information available. Empirical estimates of these measures can be straightforwardly defined. However, as, in practice, the k‐th gap‐time will not be observed for some individuals, an appropriate weighting technique should be applied for them.

To summarize, we have proposed a model for the joint evolution of a normally distributed marker and the visiting process of patients. It can provide dynamic predictions for the time‐to‐next visit, utilizing previously observed visit patterns of individuals, which are updated when the next visit of the patient becomes realization and, generally, further information becomes available. Such individualized predictions are particularly valuable in the era of precision medicine and the routine collection of electronic health records, providing clinicians with tailored insights to guide patient‐specific decision‐making.

## Funding

The authors have nothing to report.

## Conflicts of Interest

G.T. has received grants unrelated to this work, paid to her institution, and honoraria from Gilead Sciences. The rest of the authors declare no potential conflicts of interest.

## Supporting information




**Text A:** Analytic presentation of the observed score vector of the model.
**Text B:** Simulation study results.
**Table S1:** Simulation study results, where the data have been generated by the proposed model under Scenario I‐B. Fitting model (i) is correctly specified (“Correctly specified”) and (ii) ignores the longitudinal model (“Ignoring longitudinal model”).
**Table S2:** Simulation study results, where the data have been generated by the proposed model under Scenario I‐D. Fitting model (i) is correctly specified (“Correctly specified”) and (ii) ignores the longitudinal model (“Ignoring longitudinal model”).
**Table S3:** Simulation study results for personalized survival probabilities stratified by the number of previous visits, where the data have been generated by the proposed model under Scenario I‐A. Fitting model (i) is correctly specified (“Correctly specified”) and (ii) ignores the longitudinal model (“Ignoring longitudinal model”).
**Table S4:** Simulation study results for personalized survival probabilities stratified by the number of previous visits, where the data have been generated by the proposed model under Scenario I‐C. Fitting model (i) is correctly specified (“Correctly specified”) and (ii) ignores the longitudinal model (“Ignoring longitudinal model”).
**Table S5:** Simulation study results for personalized survival probabilities stratified by the number of previous visits, where the data have been generated by the proposed model under Scenario I‐B. Fitting model (i) is correctly specified (“Correctly specified”) and (ii) ignores the longitudinal model (“Ignoring longitudinal model”).
**Table S6:** Simulation study results for personalized survival probabilities stratified by the number of previous visits, where the data have been generated by the proposed model under Scenario I‐D. Fitting model (i) is correctly specified (“Correctly specified”) and (ii) ignores the longitudinal model (“Ignoring longitudinal model”).
**Table S7:** Simulation study results, where the data have been generated by the proposed model under Scenario II‐A. Fitting model (i) is correctly specified (“Correctly specified”) and (ii) ignores the longitudinal model (“Ignoring longitudinal model”).
**Table S8:** Simulation study results, where the data have been generated by the proposed model under Scenario II‐C. Fitting model (i) is correctly specified (“Correctly specified”) and (ii) ignores the longitudinal model (“Ignoring longitudinal model”).
**Table S9:** Simulation study results, where the data have been generated by the proposed model under Scenario II‐B. Fitting model (i) is correctly specified (“Correctly specified”) and (ii) ignores the longitudinal model (“Ignoring longitudinal model”).
**Table S10:** Simulation study results, where the data have been generated by the proposed model under Scenario II‐D. Fitting model (i) is correctly specified (“Correctly specified”) and (ii) ignores the longitudinal model (“Ignoring longitudinal model”).
**Table S11:** Simulation study results for personalized survival probabilities stratified by the number of previous visits, where the data have been generated by the proposed model under Scenario II‐A. Fitting model (i) is correctly specified (“Correctly specified”) and (ii) ignores the longitudinal model (“Ignoring longitudinal model”).
**Table S12:** Simulation study results for personalized survival probabilities stratified by the number of previous visits, where the data have been generated by the proposed model under Scenario II‐C. Fitting model (i) is correctly specified (“Correctly specified”) and (ii) ignores the longitudinal model (“Ignoring longitudinal model”).
**Table S13:** Simulation study results for personalized survival probabilities stratified by the number of previous visits, where the data have been generated by the proposed model under Scenario II‐B. Fitting model (i) is correctly specified (“Correctly specified”) and (ii) ignores the longitudinal model (“Ignoring longitudinal model”).
**Table S14:** Simulation study results for personalized survival probabilities stratified by the number of previous visits, where the data have been generated by the proposed model under Scenario II‐D. Fitting model (i) is correctly specified (“Correctly specified”) and (ii) ignores the longitudinal model (“Ignoring longitudinal model”).
**Table S15:** Simulation study results, where the data have been generated a joint model in which visiting process depends on markers random intercept and rate of change of the underlying marker trajectory. Fitting model (i) is the misspecified proposed joint model (“Proposed Model”), (ii) the misspecified proportional hazards model that totally ignores the longitudinal model (“Ignoring longitudinal model”) and (iii) the misspecified linear mixed model that totally ignores the visiting process (“Ignoring visiting process”).
**Table S16:** Simulation study results under Scenario II‐A with an informative terminal event, targeting approximately 5% terminal events within the study duration. The proposed model was fitted ignoring the terminal event process.
**Table S17:** Simulation study results under Scenario II‐A with an informative terminal event, targeting approximately 40% terminal events within the study duration. The proposed model was fitted ignoring the terminal event process.
**Table S18:** Simulation study results, where the data have been generated by the proposed model and the longitudinal marker is binary. Fitting model is correctly specified (“Correctly specified”).
**Text C:** Model performance in the validation sample of AMACS data.
**Table S19:** AUC and prediction error (PE) for the proposed joint model (JM) and a simpler proportional hazards model for the visiting process (VM) in the validation sample of AMACS data.

## Data Availability

The R code implementing the proposed methodology is publicly available on GitHub at https://github.com/achilleas‐st/JMvis‐dynpred.

## References

[1] A. K. Monroe , J. A. Fleishman , C. C. Voss , et al., “Assessing Antiretroviral Use During Gaps in HIV Primary Care Using Multisite Medicaid Claims and Clinical Data,” JAIDS Journal of Acquired Immune Deficiency Syndromes 76, no. 1 (2017): 82–89. 28797023 10.1097/QAI.0000000000001469 PMC5588917

[2] J. Z. Li , E. Aga , R. J. Bosch , et al., “Time to Viral Rebound After Interruption of Modern Antiretroviral Therapies,” Clinical Infectious Diseases 74, no. 5 (2022): 865–870. 34117753 10.1093/cid/ciab541 PMC8906742

[3] C. Thomadakis , C. T. Yiannoutsos , N. Pantazis , et al., “The Effect of HIV Treatment Interruption on Subsequent Immunological Response,” American Journal of Epidemiology 192, no. 7 (2023): 1181–1191. 37045803 10.1093/aje/kwad076 PMC10326612

[4] D. Rizopoulos , Joint Models for Longitudinal and Time‐to‐Event Data: With Applications in R (CRC press, 2012).

[5] G. Papageorgiou , K. Mauff , A. Tomer , and D. Rizopoulos , “An Overview of Joint Modeling of Time‐to‐Event and Longitudinal Outcomes,” Annual Review of Statistics and Its Application 6, no. 1 (2019): 223–240.

[6] G. Touloumi , S. Pocock , A. Babiker , and J. Darbyshire , “Estimation and Comparison of Rates of Change in Longitudinal Studies With Informative Drop‐Outs,” Statistics in Medicine 18, no. 10 (1999): 1215–1233. 10363341 10.1002/(sici)1097-0258(19990530)18:10<1215::aid-sim118>3.0.co;2-6

[7] R. L. Prentice , “Covariate Measurement Errors and Parameter Estimation in a Failure Time Regression Model,” Biometrika 69, no. 2 (1982): 331–342.

[8] E. M. Pullenayegum and L. S. Lim , “Longitudinal Data Subject to Irregular Observation: A Review of Methods With a Focus on Visit Processes, Assumptions, and Study Design,” Statistical Methods in Medical Research 25, no. 6 (2016): 2992–3014. 24855119 10.1177/0962280214536537

[9] D. B. Rubin , “Inference and Missing Data,” Biometrika 63, no. 3 (1976): 581–592.

[10] Y. Liang , W. Lu , and Z. Ying , “Joint Modeling and Analysis of Longitudinal Data With Informative Observation Times,” Biometrics 65, no. 2 (2009): 377–384. 18759841 10.1111/j.1541-0420.2008.01104.x

[11] L. Sun , X. Song , J. Zhou , and L. Liu , “Joint Analysis of Longitudinal Data With Informative Observation Times and a Dependent Terminal Event,” Journal of the American Statistical Association 107, no. 498 (2012): 688–700.

[12] L. Liu , X. Huang , and J. O'Quigley , “Analysis of Longitudinal Data in the Presence of Informative Observational Times and a Dependent Terminal Event, With Application to Medical Cost Data,” Biometrics 64, no. 3 (2008): 950–958. 18162110 10.1111/j.1541-0420.2007.00954.x

[13] L. Zhu , H. Zhao , J. Sun , S. Pounds , and H. Zhang , “Joint Analysis of Longitudinal Data and Recurrent Episodes Data With Application to Medical Cost Analysis,” Biometrical Journal 55, no. 1 (2013): 5–16. 23225670 10.1002/bimj.201200018

[14] C. Thomadakis , L. Meligkotsidou , N. Pantazis , and G. Touloumi , “Shared Parameter Modeling of Longitudinal Data Allowing for Possibly Informative Visiting Process and Terminal Event,” Biostatistics 26 (2024): kxae041. 10.1093/biostatistics/kxae041 PMC11911807 39449049

[15] P. M. Afonso , D. Rizopoulos , A. K. Palipana , et al., “A Joint Model for (Un) Bounded Longitudinal Markers, Competing Risks, and Recurrent Events Using Patient Registry Data,” Statistics in Medicine 44, no. 8–9 (2025): e70057. 40277342 10.1002/sim.70057 PMC12023843

[16] D. Rizopoulos , “Dynamic Predictions and Prospective Accuracy in Joint Models for Longitudinal and Time‐To‐Event Data,” Biometrics 67, no. 3 (2011): 819–829. 21306352 10.1111/j.1541-0420.2010.01546.x

[17] C. Proust‐Lima and J. M. Taylor , “Development and Validation of a Dynamic Prognostic Tool for Prostate Cancer Recurrence Using Repeated Measures of Posttreatment PSA: A Joint Modeling Approach,” Biostatistics 10, no. 3 (2009): 535–549. 19369642 10.1093/biostatistics/kxp009 PMC2697347

[18] J. Musoro , G. Struijk , R. Geskus , I. Ten Berge , and A. Zwinderman , “Dynamic Prediction of Recurrent Events Data by Landmarking With Application to a Follow‐Up Study of Patients After Kidney Transplant,” Statistical Methods in Medical Research 27, no. 3 (2018): 832–845. 27142981 10.1177/0962280216643563

[19] I. Yokota and Y. Matsuyama , “Dynamic Prediction of Repeated Events Data Based on Landmarking Model: Application to Colorectal Liver Metastases Data,” BMC Medical Research Methodology 19 (2019): 1–11. 30764772 10.1186/s12874-019-0677-0 PMC6376774

[20] S. Serrano‐Villar , T. Sainz , S. A. Lee , et al., “HIV‐Infected Individuals With Low CD4/CD8 Ratio Despite Effective Antiretroviral Therapy Exhibit Altered T Cell Subsets, Heightened CD8+ T Cell Activation, and Increased Risk of Non‐AIDS Morbidity and Mortality,” PLoS Pathogens 10, no. 5 (2014): e1004078. 24831517 10.1371/journal.ppat.1004078 PMC4022662

[21] A. Genz and F. Bretz , Computation of Multivariate Normal and t Probabilities. Lecture Notes in StatisticsHeidelberg (Springer‐Verlag, 2009).

[22] N. Pantazis , V. Papastamopoulos , V. Paparizos , et al., “Long‐Term Evolution of CD4+ Cell Count in Patients Under Combined Antiretroviral Therapy,” AIDS 33, no. 10 (2019): 1645–1655. 31305332 10.1097/QAD.0000000000002248

[23] D. Zhang , M. H. Chen , J. G. Ibrahim , M. E. Boye , P. Wang , and W. Shen , “Assessing Model Fit in Joint Models of Longitudinal and Survival Data With Applications to Cancer Clinical Trials,” Statistics in Medicine 33, no. 27 (2014): 4715–4733. 25044061 10.1002/sim.6269 PMC4221436

[24] A. Charles‐Nelson , S. Katsahian , and C. Schramm , “How to Analyze and Interpret Recurrent Events Data in the Presence of a Terminal Event: An Application on Readmission After Colorectal Cancer Surgery,” Statistics in Medicine 38, no. 18 (2019): 3476–3502. 31016792 10.1002/sim.8168

[25] L. D. Amorim and J. Cai , “Modelling Recurrent Events: A Tutorial for Analysis in Epidemiology,” International Journal of Epidemiology 44, no. 1 (2015): 324–333. 25501468 10.1093/ije/dyu222 PMC4339761

[26] D. Rizopoulos , G. Molenberghs , and E. M. Lesaffre , “Dynamic Predictions With Time‐Dependent Covariates in Survival Analysis Using Joint Modeling and Landmarking,” Biometrical Journal 59, no. 6 (2017): 1261–1276. 28792080 10.1002/bimj.201600238

[27] P. Blanche , C. Proust‐Lima , L. Loubère , C. Berr , J. F. Dartigues , and H. Jacqmin‐Gadda , “Quantifying and Comparing Dynamic Predictive Accuracy of Joint Models for Longitudinal Marker and Time‐To‐Event in Presence of Censoring and Competing Risks,” Biometrics 71, no. 1 (2015): 102–113. 25311240 10.1111/biom.12232

