# Supplementary material for: Joint Modeling of Longitudinal Data and Potentially Informative Visiting Process to Dynamically Predict Future Visit Times
Source: Stat Med. 2026 Aug 9;45(18-19):e70702. doi: 10.1002/sim.70702 (PMC13454517; doi:10.1002/sim.70702)
Supplement: Supplementary file 1 [file SIM-45-0-s001.pdf]

Received: Added at production | Revised: Added at production | Accepted: Added at production

DOI: xxx/xxxx

ARTICLE TYPE

Joint modeling of longitudinal data and potentially informative visiting process to dynamically predict future visit times

Achilleas Stamoulopoulos[1] | Christos Thomadakis[1,2] | Giota Touloumi[1]

[1]Department of Hygiene, Epidemiology and Medical Statistics, Medical School, National and Kapodistrian University of Athens, Mikras Asias 75, Athens, 115 27, Greece

[2]Department of Statistics, Athens University of Economics and Business, Patission 76, Athens, 104 34, Greece

Correspondence
Achilleas Stamoulopoulos.
Email: achilleasst@med.uoa.gr

Present address
Department of Hygiene, Epidemiology and Medical Statistics, Medical School, National and Kapodistrian University of Athens, Mikras Asias 75, Athens, 115 27, Greece.

Abstract

This document is the supplementary material for the paper entitled "Joint modeling of longitudinal data and potentially informative visiting process to dynamically predict future visit times". In Web Appendix A, an analytic presentation of the observed score vector of the proposed model is given. In Web Appendix B, additional results from the conducted simulation studies are provided. Web Appendix C presents the performance measures (AUC and prediction error) of both the proposed joint model and the simpler proportional hazards model, evaluated in the validation sample of the AMACS data.

KEYWORDS

joint modeling, informative visiting, dynamic predictions, prediction of future visit times

1 | WEB APPENDIX A: ANALYTIC PRESENTATION OF THE OBSERVED SCORE VECTOR OF THE MODEL

The observed score vector for the ith individual is

$$\begin{aligned}
S_i(\boldsymbol{\theta}) &= \frac{\partial}{\partial \boldsymbol{\theta}} \log \int f(\boldsymbol{w}_i|u_i, \boldsymbol{Y}_i; \boldsymbol{\theta}) f(u_i; \boldsymbol{\theta}) f(\boldsymbol{Y}_i|u_i; \boldsymbol{\theta})\, du_i \\
&= \frac{1}{f(\boldsymbol{w}_i, \boldsymbol{Y}_i; \boldsymbol{\theta})} \frac{\partial}{\partial \boldsymbol{\theta}} \int f(\boldsymbol{w}_i|u_i, \boldsymbol{Y}_i; \boldsymbol{\theta}) f(u_i; \boldsymbol{\theta}) f(\boldsymbol{Y}_i|u_i; \boldsymbol{\theta})\, du_i \\
&= \frac{1}{f(\boldsymbol{w}_i, \boldsymbol{Y}_i; \boldsymbol{\theta})} \int \frac{\partial}{\partial \boldsymbol{\theta}} \{ f(\boldsymbol{w}_i|u_i, \boldsymbol{Y}_i; \boldsymbol{\theta}) f(u_i; \boldsymbol{\theta}) f(\boldsymbol{Y}_i|u_i; \boldsymbol{\theta}) \}\, du_i \\
&= \int \left[\frac{\partial}{\partial \boldsymbol{\theta}} \log\{ f(\boldsymbol{w}_i|u_i, \boldsymbol{Y}_i; \boldsymbol{\theta}) f(u_i; \boldsymbol{\theta}) f(\boldsymbol{Y}_i|u_i; \boldsymbol{\theta}) \} \right] \times \frac{f(\boldsymbol{w}_i|u_i, \boldsymbol{Y}_i; \boldsymbol{\theta}) f(u_i; \boldsymbol{\theta}) f(\boldsymbol{Y}_i|u_i; \boldsymbol{\theta})}{f(\boldsymbol{w}_i, \boldsymbol{Y}_i; \boldsymbol{\theta})}\, du_i \\
&= \int A(\boldsymbol{\theta}, u_i) \times \frac{f(\boldsymbol{w}_i|u_i, \boldsymbol{Y}_i; \boldsymbol{\theta}) f(u_i; \boldsymbol{\theta}) f(\boldsymbol{Y}_i|u_i; \boldsymbol{\theta})}{f(\boldsymbol{w}_i, \boldsymbol{Y}_i; \boldsymbol{\theta})}\, du_i \qquad (1)
\end{aligned}$$

where $A(\boldsymbol{\theta}, u_i)$ is the complete data score vector. Below, we present the formulas for the calculation of the complete data score vector. Based on them, the observed data score vector can be approximated using Gauss-Hermite quadrature.

 © 2023 Copyright Holder Name

Visiting model parameters (2.1), $\boldsymbol{\theta}_v$ (omitting the σ_u^2 from the components of the vector, as the formula for the complete data score vector with respect to the components of matrix $\boldsymbol{D}$ will be given separately), appear only in $f(\boldsymbol{w}_i|u_i, \boldsymbol{Y}_i; \boldsymbol{\theta})$ part of $A(\boldsymbol{\theta}, u_i)$. Hence, we obtain

$$\frac{\partial \log f(\boldsymbol{w}_i|u_i, \boldsymbol{Y}_i; \boldsymbol{\theta}_v)}{\partial \boldsymbol{\theta}_v} = \sum_{j=1}^{n_i-1} \begin{bmatrix} \boldsymbol{I}(w_{ij}) \\ \overline{\boldsymbol{X}}_i(t_{ij}) \\ \boldsymbol{g}\{\overline{\boldsymbol{Y}}_i(t_{ij})\} \end{bmatrix} - \sum_{j=1}^{n_i} \int_0^{w_{ij}} \begin{bmatrix} \boldsymbol{I}(s) \\ \overline{\boldsymbol{X}}_i(t_{ij}) \\ \boldsymbol{g}\{\overline{\boldsymbol{Y}}_i(t_{ij})\} \end{bmatrix} \psi_{ij}(\boldsymbol{\theta}_v, u_i)\, ds, \tag{2}$$

where $\psi_{ij}(\boldsymbol{\theta}_v, u_i) = \exp\left[\boldsymbol{\eta}^\top \boldsymbol{I}(s) + \boldsymbol{\phi}^\top \overline{\boldsymbol{X}}_i(t_{ij}) + \boldsymbol{\gamma}^\top \boldsymbol{g}\{\overline{\boldsymbol{Y}}_i(t_{ij})\} + u_i\right]$, $\boldsymbol{\eta}^\top = (\eta_1, \eta_2, \ldots, \eta_{K+1})$, $\eta_k = \log(\zeta_k), k = 1, \ldots, K+1$.

Longitudinal model parameters, $\boldsymbol{\theta}_l$ (again, omitting the components of $\boldsymbol{D}_b$ from the vector), appear only in $f(\boldsymbol{Y}_i|u_i; \boldsymbol{\theta})$ part of $A(\boldsymbol{\theta}, u_i)$. Letting $\xi = \log(\sigma_\epsilon)$, we obtain

$$\frac{\partial \log f(\boldsymbol{Y}_i|u_i; \boldsymbol{\theta})}{\partial \xi} = \exp(2\xi)\left\{(\boldsymbol{Y}_i - \boldsymbol{\mu}_{iY|u})^\top \boldsymbol{\Sigma}_{iY|u}^{-1} \boldsymbol{\Sigma}_{iY|u}^{-1} (\boldsymbol{Y}_i - \boldsymbol{\mu}_{iY|u}) - \mathrm{Tr}\left(\boldsymbol{\Sigma}_{iY|u}^{-1}\right)\right\} \tag{3}$$

and

$$\frac{\partial \log f(\boldsymbol{Y}_i|u_i; \boldsymbol{\theta})}{\partial \boldsymbol{\beta}} = \boldsymbol{X}_i^\top \boldsymbol{\Sigma}_{iY|u}^{-1} (\boldsymbol{Y}_i - \boldsymbol{\mu}_{iY|u}) \tag{4}$$

For the parameters of the variance-covariance matrix $\boldsymbol{D} = \begin{pmatrix} \sigma_u^2 & \boldsymbol{D}_{ub} \\ * & \boldsymbol{D}_b \end{pmatrix}$, we denote with $\boldsymbol{L} = \begin{pmatrix} l_{11} & \boldsymbol{0} \\ \boldsymbol{L}_{21} & \boldsymbol{L}_{22} \end{pmatrix}$ the Cholesky factor of it. It follows that $\sigma_u^2 = l_{11}^2$, $\boldsymbol{D}_{ub}^\top = l_{11}\boldsymbol{L}_{21}$ and $\boldsymbol{D}_b = \boldsymbol{L}_{21}\boldsymbol{L}_{21}^\top + \boldsymbol{L}_{22}\boldsymbol{L}_{22}^\top$. Hence, the conditional mean and variance-covariance matrix of $\boldsymbol{Y}_i$ given u_i can be written as $\boldsymbol{\mu}_{iY|u} = \boldsymbol{X}_i\boldsymbol{\beta} + \frac{u_i}{l_{11}}\boldsymbol{Z}_i\boldsymbol{L}_{21}$ and $\boldsymbol{\Sigma}_{iY|u} = \sigma_\epsilon^2 \boldsymbol{I}_{n_i} + \boldsymbol{Z}_i \boldsymbol{L}_{22} \boldsymbol{L}_{22}^\top \boldsymbol{Z}_i^\top$. l_{11} appears on both $f(u_i; \boldsymbol{\theta})$ and $f(\boldsymbol{Y}_i|u_i; \boldsymbol{\theta})$, while $\boldsymbol{L}_{21}$ and $\boldsymbol{L}_{22}$ appear only on $f(\boldsymbol{Y}_i|u_i; \boldsymbol{\theta})$. So, we obtain

$$\frac{\partial \log\{f(u_i; \boldsymbol{\theta}) f(\boldsymbol{Y}_i|u_i; \boldsymbol{\theta})\}}{\partial l_{11}} = -\frac{1}{l_{11}} + \frac{u_i^2}{l_{11}^3} - \frac{u_i}{l_{11}^2} \boldsymbol{L}_{21}^\top \boldsymbol{Z}_i^\top \boldsymbol{\Sigma}_{iY|u}^{-1} (\boldsymbol{Y}_i - \boldsymbol{\mu}_{iY|u}), \tag{5}$$

$$\frac{\partial \log f(\boldsymbol{Y}_i|u_i; \boldsymbol{\theta})}{\partial \boldsymbol{L}_{21}} = \frac{u_i}{l_{11}} \boldsymbol{Z}_i^\top \boldsymbol{\Sigma}_{iY|u}^{-1} (\boldsymbol{Y}_i - \boldsymbol{\mu}_{iY|u}), \tag{6}$$

and

$$\frac{\partial \log f(\boldsymbol{Y}_i|u_i; \boldsymbol{\theta})}{\partial L_{22,pq}} = \frac{1}{2}\left\{(\boldsymbol{Y}_i - \boldsymbol{\mu}_{iY|u})^\top \boldsymbol{\Sigma}_{iY|u}^{-1} \frac{\partial \boldsymbol{\Sigma}_{iY|u}}{\partial L_{22,pq}} \boldsymbol{\Sigma}_{iY|u}^{-1} (\boldsymbol{Y}_i - \boldsymbol{\mu}_{iY|u}) - \mathrm{Tr}\left(\boldsymbol{\Sigma}_{iY|u}^{-1} \frac{\partial \boldsymbol{\Sigma}_{iY|u}}{\partial L_{22,pq}}\right)\right\} \tag{7}$$

for $p \geq q$, where $\frac{\partial \boldsymbol{\Sigma}_{iY|u}}{\partial L_{22,pq}} = \boldsymbol{Z}_i(\boldsymbol{E}_{pq}\boldsymbol{L}_{22}^\top + \boldsymbol{L}_{22}\boldsymbol{E}_{qp})\boldsymbol{Z}_i^\top$ and $\boldsymbol{E}_{pq}$ the square matrix with 1 in the (p, q)-entry and 0 elsewhere, of dimensions equal to the dimensions of $\boldsymbol{L}_{22}$. Note, here, that for l_{11} and the diagonal components of $\boldsymbol{L}_{22}$, we reparameterize $\boldsymbol{D}$ considering the natural logarithm of these quantities (i.e. log-Cholesky parameterization), hence we use the chain rule before applying the above formulas.

2 | WEB APPENDIX B: SIMULATION STUDY RESULTS

Additional simulation results corresponding to the studies described in Section 3.2 of the main manuscript are presented below. Specifically, we complete the results for the scenarios with less frequent patient visits (Scenarios I-B and I-D), provide the results for the scenarios with more frequent visits (Scenarios II-A, II-B, II-C, and II-D), report the results for the scenario generated under the alternative joint model scenario and the scenarios where the underlying data generation mechanism involves a terminal event which is ignored in the fitting procedure, and, finally, present the results for the correctly specified model in which the longitudinal marker is binary.

2.1 | Less-frequent patient visits scenarios

TABLE 1 Simulation study results, where the data have been generated by the proposed model under Scenario I-B. Fitting model (i) is correctly specified ("Correctly specified") and (ii) ignores the longitudinal model ("Ignoring longitudinal model").

Parameter	Correctly Specified							Ignoring longitudinal model					
	**True**	**Est**	**Bias**	**MSE**	**ASE**	**MCSD**	**Cov.**	**Est**	**Bias**	**MSE**	**ASE**	**MCSD**	**Cov.**
**Longitudinal**													
Intercept (β_0)	17.50	17.529	0.029	0.138	0.382	0.371	95.6	-	-	-	-	-	-
t (β_1)	-1.10	-1.096	0.004	0.003	0.052	0.050	95.6	-	-	-	-	-	-
$\sqrt{t}$ (β_2)	6.30	6.289	-0.011	0.038	0.199	0.195	95.6	-	-	-	-	-	-
V_1 (β_3)	1.50	1.475	-0.025	0.215	0.469	0.465	94.2	-	-	-	-	-	-
**Visiting Process**													
Observed marker value (γ)	-0.10	-0.100	0.000	0.000	0.004	0.004	96.4	-0.090	0.010	0.000	0.004	0.004	28.1
V_2 (ϕ)	0.50	0.488	-0.012	0.021	0.141	0.145	94.8	0.489	-0.011	0.026	0.156	0.161	93.0
**Variance components**													
σ_u^2	3	2.995	-0.005	0.042	0.210	0.205	95.6	2.902	-0.098	0.040	0.204	0.201	91.8
ρ_{u,b_0}	0.25	0.250	0.000	0.002	0.045	0.046	93.6	-	-	-	-	-	-
ρ_{u,b_1}	-0.2	0.199	0.001	0.003	0.052	0.052	95.2	-	-	-	-	-	-
ρ_{u,b_2}	0.25	0.250	0.000	0.002	0.049	0.050	95.0	-	-	-	-	-	-
**Personalized survival probabilities**													
$\Pr(w_k > 6 \text{ months} \mid \boldsymbol{w}, \boldsymbol{Y})$	-	-	0.4%	-	-	-	96.0	-	0.8%	-	-	-	42.7
$\Pr(w_k > 12 \text{ months} \mid \boldsymbol{w}, \boldsymbol{Y})$	-	-	0.6%	-	-	-	95.4	-	1.3%	-	-	-	42.5
$\Pr(w_k > 18 \text{ months} \mid \boldsymbol{w}, \boldsymbol{Y})$	-	-	0.5%	-	-	-	95.6	-	1.3%	-	-	-	42.9
$\Pr(w_k > 24 \text{ months} \mid \boldsymbol{w}, \boldsymbol{Y})$	-	-	0.5%	-	-	-	95.2	-	1.3%	-	-	-	42.5

Results from 500 replications with each dataset including 500 individuals. "True" denotes the true parameter values; "Est" the mean of the estimates over the 500 replications; "Bias" the mean bias of estimates; "MSE" the mean squared error; "ASE" the average SE; "MCSD" the empirical Monte Carlo deviation of estimates and "Cov." the empirical coverage probability (%) of the confidence intervals. For the Personalized survival probabilities–where a global true values cannot be defined as they depend on specific patients–, "Bias" indicates the mean absolute deviation between the true, patient-specific probability and the estimated one, while "Cov." the proportion of simulations in which the estimated confidence interval contained the true, patient-specific probability.

TABLE 2 Simulation study results, where the data have been generated by the proposed model under Scenario I-D. Fitting model (i) is correctly specified ("Correctly specified") and (ii) ignores the longitudinal model ("Ignoring longitudinal model").

Parameter	Correctly Specified							Ignoring longitudinal model					
	**True**	**Est**	**Bias**	**MSE**	**ASE**	**MCSD**	**Cov.**	**Est**	**Bias**	**MSE**	**ASE**	**MCSD**	**Cov.**
**Longitudinal**													
Intercept (β_0)	17.50	17.464	-0.036	0.147	0.374	0.384	94.2	-	-	-	-	-	-
t (β_1)	-1.10	-1.101	-0.001	0.003	0.052	0.051	95.4	-	-	-	-	-	-
$\sqrt{t}$ (β_2)	6.30	6.307	0.007	0.036	0.200	0.191	94.4	-	-	-	-	-	-
V_1 (β_3)	1.50	1.537	0.037	0.178	0.443	0.423	96.0	-	-	-	-	-	-
**Visiting Process**													
Observed marker value (γ)	-0.10	-0.100	0.000	0.000	0.004	0.004	95.4	-0.088	0.012	0.000	0.004	0.004	12.8
V_2 (ϕ)	0.50	0.493	-0.007	0.015	0.126	0.124	93.6	0.490	-0.010	0.023	0.155	0.152	95.6
**Variance components**													
σ_u^2	3	3.001	0.001	0.046	0.212	0.214	95.4	2.871	-0.129	0.042	0.203	0.206	88.2
ρ_{u,b_0}	0.45	0.451	0.001	0.002	0.039	0.040	94.0	-	-	-	-	-	-
ρ_{u,b_1}	-0.40	-0.401	-0.001	0.002	0.046	0.046	95.2	-	-	-	-	-	-
ρ_{u,b_2}	0.25	0.251	0.001	0.003	0.049	0.052	93.4	-	-	-	-	-	-
**Personalized survival probabilities**													
$\Pr(w_k > 6 \text{ months} \mid \boldsymbol{w}, \boldsymbol{Y})$	-	-	0.3%	-	-	-	94.4	-	0.9%	-	-	-	31.9
$\Pr(w_k > 12 \text{ months} \mid \boldsymbol{w}, \boldsymbol{Y})$	-	-	0.5%	-	-	-	93.4	-	1.7%	-	-	-	32.5
$\Pr(w_k > 18 \text{ months} \mid \boldsymbol{w}, \boldsymbol{Y})$	-	-	0.5%	-	-	-	93.6	-	1.8%	-	-	-	32.7
$\Pr(w_k > 24 \text{ months} \mid \boldsymbol{w}, \boldsymbol{Y})$	-	-	0.5%	-	-	-	93.6	-	1.9%	-	-	-	32.9

Results from 500 replications with each dataset including 500 individuals. "True" denotes the true parameter values; "Est" the mean of the estimates over the 500 replications; "Bias" the mean bias of estimates; "MSE" the mean squared error; "ASE" the average SE; "MCSD" the empirical Monte Carlo deviation of estimates and "Cov." the empirical coverage probability (%) of the confidence intervals. For the Personalized survival probabilities (where global true values cannot be defined as they depend on specific patients), "Bias" indicates the mean absolute deviation between the true, patient-specific probability and the estimated one, while "Cov." the proportion of simulations in which the estimated confidence interval contained the true, patient-specific probability.

TABLE 3 Simulation study results for personalized survival probabilities stratified by the number of previous visits, where the data have been generated by the proposed model under Scenario I-A. Fitting model (i) is correctly specified ("Correctly specified") and (ii) ignores the longitudinal model ("Ignoring longitudinal model").

**Parameter**	**Correctly Specified**		**Ignoring longitudinal model**	
	**Bias**	**Cov.**	**Bias**	**Cov.**
**Personalized survival probabilities**				
$k \leq 7$				
$\Pr(w_k > 6 \text{ months}\vert \boldsymbol{w}, \boldsymbol{Y})$	0.1%	95.1	0.2%	42.9
$\Pr(w_k > 12 \text{ months}\vert \boldsymbol{w}, \boldsymbol{Y})$	0.3%	95.1	1.0%	41.3
$\Pr(w_k > 18 \text{ months}\vert \boldsymbol{w}, \boldsymbol{Y})$	0.6%	94.6	1.7%	40.8
$\Pr(w_k > 24 \text{ months}\vert \boldsymbol{w}, \boldsymbol{Y})$	0.7%	94.6	2.1%	41.3
$7 < k \leq 12$				
$\Pr(w_k > 6 \text{ months}\vert \boldsymbol{w}, \boldsymbol{Y})$	0.1%	98.5	0.5%	31.8
$\Pr(w_k > 12 \text{ months}\vert \boldsymbol{w}, \boldsymbol{Y})$	0.4%	99.0	2.0%	31.8
$\Pr(w_k > 18 \text{ months}\vert \boldsymbol{w}, \boldsymbol{Y})$	0.5%	99.0	2.5%	31.8
$\Pr(w_k > 24 \text{ months}\vert \boldsymbol{w}, \boldsymbol{Y})$	0.6%	99.0	2.7%	32.8
$k > 12$				
$\Pr(w_k > 6 \text{ months}\vert \boldsymbol{w}, \boldsymbol{Y})$	0.9%	96.6	3.0%	7.6
$\Pr(w_k > 12 \text{ months}\vert \boldsymbol{w}, \boldsymbol{Y})$	0.7%	98.3	3.1%	9.2
$\Pr(w_k > 18 \text{ months}\vert \boldsymbol{w}, \boldsymbol{Y})$	0.4%	95.0	2.2%	10.9
$\Pr(w_k > 24 \text{ months}\vert \boldsymbol{w}, \boldsymbol{Y})$	0.3%	95.8	1.5%	7.6

"Bias" indicates the mean absolute deviation between the true, patient-specific probability and the estimated one, while "Cov." the proportion of simulations in which the estimated confidence interval contained the true, patient-specific probability, k denotes the number of previous visits.

TABLE 4 Simulation study results for personalized survival probabilities stratified by the number of previous visits, where the data have been generated by the proposed model under Scenario I-C. Fitting model (i) is correctly specified ("Correctly specified") and (ii) ignores the longitudinal model ("Ignoring longitudinal model").

Parameter	Correctly Specified		Ignoring longitudinal model	
	**Bias**	**Cov.**	**Bias**	**Cov.**
**Personalized survival probabilities**				
$k \leq 7$				
$\Pr(w_k > 6 \text{ months} \mid \boldsymbol{w}, \boldsymbol{Y})$	0.1%	93.6	0.6%	8.9
$\Pr(w_k > 12 \text{ months} \mid \boldsymbol{w}, \boldsymbol{Y})$	0.5%	93.0	3.2%	8.3
$\Pr(w_k > 18 \text{ months} \mid \boldsymbol{w}, \boldsymbol{Y})$	0.8%	93.0	5.2%	7.0
$\Pr(w_k > 24 \text{ months} \mid \boldsymbol{w}, \boldsymbol{Y})$	1.0%	93.6	6.7%	8.3
$7 < k \leq 12$				
$\Pr(w_k > 6 \text{ months} \mid \boldsymbol{w}, \boldsymbol{Y})$	0.2%	96.1	1.2%	15.4
$\Pr(w_k > 12 \text{ months} \mid \boldsymbol{w}, \boldsymbol{Y})$	0.8%	96.9	4.7%	16.2
$\Pr(w_k > 18 \text{ months} \mid \boldsymbol{w}, \boldsymbol{Y})$	1.0%	96.1	6.0%	16.7
$\Pr(w_k > 24 \text{ months} \mid \boldsymbol{w}, \boldsymbol{Y})$	1.1%	96.1	6.2%	15.8
$k > 12$				
$\Pr(w_k > 6 \text{ months} \mid \boldsymbol{w}, \boldsymbol{Y})$	1.3%	93.9	8.1%	0.0
$\Pr(w_k > 12 \text{ months} \mid \boldsymbol{w}, \boldsymbol{Y})$	1.8%	94.8	12.2%	1.7
$\Pr(w_k > 18 \text{ months} \mid \boldsymbol{w}, \boldsymbol{Y})$	1.4%	94.8	9.4%	1.7
$\Pr(w_k > 24 \text{ months} \mid \boldsymbol{w}, \boldsymbol{Y})$	1.0%	95.7	6.8%	1.7

"Bias" indicates the mean absolute deviation between the true, patient-specific probability and the estimated one, while "Cov." the proportion of simulations in which the estimated confidence interval contained the true, patient-specific probability, k denotes the number of previous visits.

TABLE 5 Simulation study results for personalized survival probabilities stratified by the number of previous visits, where the data have been generated by the proposed model under Scenario I-B. Fitting model (i) is correctly specified ("Correctly specified") and (ii) ignores the longitudinal model ("Ignoring longitudinal model").

**Parameter**	**Correctly Specified**		**Ignoring longitudinal model**	
	**Bias**	**Cov.**	**Bias**	**Cov.**
**Personalized survival probabilities**				
$k \leq 7$				
$\Pr(w_k > 6 \text{ months} \mid \boldsymbol{w}, \boldsymbol{Y})$	0.1%	95.9	0.1%	55.3
$\Pr(w_k > 12 \text{ months} \mid \boldsymbol{w}, \boldsymbol{Y})$	0.5%	95.9	0.9%	55.9
$\Pr(w_k > 18 \text{ months} \mid \boldsymbol{w}, \boldsymbol{Y})$	0.5%	95.9	1.0%	54.7
$\Pr(w_k > 24 \text{ months} \mid \boldsymbol{w}, \boldsymbol{Y})$	0.6%	96.5	1.3%	54.7
$7 < k \leq 12$				
$\Pr(w_k > 6 \text{ months} \mid \boldsymbol{w}, \boldsymbol{Y})$	0.1%	96.8	0.4%	46.8
$\Pr(w_k > 12 \text{ months} \mid \boldsymbol{w}, \boldsymbol{Y})$	0.5%	96.3	1.3%	45.7
$\Pr(w_k > 18 \text{ months} \mid \boldsymbol{w}, \boldsymbol{Y})$	0.7%	95.7	1.7%	45.7
$\Pr(w_k > 24 \text{ months} \mid \boldsymbol{w}, \boldsymbol{Y})$	0.7%	96.3	1.8%	45.7
$k > 12$				
$\Pr(w_k > 6 \text{ months} \mid \boldsymbol{w}, \boldsymbol{Y})$	1.2%	95.0	2.4%	22.0
$\Pr(w_k > 12 \text{ months} \mid \boldsymbol{w}, \boldsymbol{Y})$	0.9%	93.6	1.8%	22.0
$\Pr(w_k > 18 \text{ months} \mid \boldsymbol{w}, \boldsymbol{Y})$	0.4%	95.0	1.1%	24.8
$\Pr(w_k > 24 \text{ months} \mid \boldsymbol{w}, \boldsymbol{Y})$	0.2%	92.2	0.7%	23.4

Bias" indicates the mean absolute deviation between the true, patient-specific probability and the estimated one, while Cov." the proportion of simulations in which the estimated confidence interval contained the true, patient-specific probability, k denotes the number of previous visits.

TABLE 6 Simulation study results for personalized survival probabilities stratified by the number of previous visits, where the data have been generated by the proposed model under Scenario I-D. Fitting model (i) is correctly specified ("Correctly specified") and (ii) ignores the longitudinal model ("Ignoring longitudinal model").

**Parameter**	**Correctly Specified**		**Ignoring longitudinal model**	
	**Bias**	**Cov.**	**Bias**	**Cov.**
**Personalized survival probabilities**				
$k \leq 7$				
$\Pr(w_k > 6 \text{ months} \mid \boldsymbol{w}, \boldsymbol{Y})$	0.1%	91.8	0.2%	46.7
$\Pr(w_k > 12 \text{ months} \mid \boldsymbol{w}, \boldsymbol{Y})$	0.6%	92.3	1.0%	48.2
$\Pr(w_k > 18 \text{ months} \mid \boldsymbol{w}, \boldsymbol{Y})$	0.5%	92.8	1.3%	48.2
$\Pr(w_k > 24 \text{ months} \mid \boldsymbol{w}, \boldsymbol{Y})$	0.6%	92.8	1.6%	47.2
$7 < k \leq 12$				
$\Pr(w_k > 6 \text{ months} \mid \boldsymbol{w}, \boldsymbol{Y})$	0.1%	94.4	0.6%	27.2
$\Pr(w_k > 12 \text{ months} \mid \boldsymbol{w}, \boldsymbol{Y})$	0.5%	91.7	2.2%	27.2
$\Pr(w_k > 18 \text{ months} \mid \boldsymbol{w}, \boldsymbol{Y})$	0.6%	92.2	2.7%	27.2
$\Pr(w_k > 24 \text{ months} \mid \boldsymbol{w}, \boldsymbol{Y})$	0.7%	92.2	2.9%	27.2
$k > 12$				
$\Pr(w_k > 6 \text{ months} \mid \boldsymbol{w}, \boldsymbol{Y})$	0.8%	98.4	2.4%	15.3
$\Pr(w_k > 12 \text{ months} \mid \boldsymbol{w}, \boldsymbol{Y})$	0.5%	97.6	2.0%	15.3
$\Pr(w_k > 18 \text{ months} \mid \boldsymbol{w}, \boldsymbol{Y})$	0.3%	96.8	1.4%	16.1
$\Pr(w_k > 24 \text{ months} \mid \boldsymbol{w}, \boldsymbol{Y})$	0.2%	96.8	0.9%	18.5

"Bias" indicates the mean absolute deviation between the true, patient-specific probability and the estimated one, while "Cov." the proportion of simulations in which the estimated confidence interval contained the true, patient-specific probability, k denotes the number of previous visits.

2.2 | Frequent patient visits scenarios

TABLE 7 Simulation study results, where the data have been generated by the proposed model under Scenario II-A. Fitting model (i) is correctly specified ("Correctly specified") and (ii) ignores the longitudinal model ("Ignoring longitudinal model").

Parameter	Correctly Specified							Ignoring longitudinal model					
	**True**	**Est**	**Bias**	**MSE**	**ASE**	**MCSD**	**Cov.**	**Est**	**Bias**	**MSE**	**ASE**	**MCSD**	**Cov.**
**Longitudinal**													
Intercept (β_0)	17.50	17.518	0.018	0.144	0.357	0.380	93.4	-	-	-	-	-	-
t (β_1)	-1.10	-1.097	0.003	0.003	0.050	0.051	95.0	-	-	-	-	-	-
$\sqrt{t}$ (β_2)	6.30	6.285	-0.015	0.039	0.194	0.199	94.0	-	-	-	-	-	-
V_1 (β_3)	1.50	1.486	-0.014	0.177	0.392	0.422	93.0	-	-	-	-	-	-
**Visiting Process**													
Observed marker value (γ)	-0.10	-0.100	0.000	0.000	0.003	0.003	94.4	-0.089	0.011	0.000	0.003	0.003	11.0
V_2 (ϕ)	0.50	0.493	-0.007	0.012	0.117	0.111	96.4	0.500	0.000	0.021	0.153	0.144	95.6
**Variance components**													
σ_u^2	3	2.986	-0.014	0.041	0.205	0.204	94.6	2.848	-0.152	0.040	0.196	0.201	86.8
ρ_{u,b_0}	0.45	0.447	-0.003	0.002	0.038	0.039	93.6	-	-	-	-	-	-
ρ_{u,b_1}	-0.20	-0.200	0.000	0.003	0.050	0.050	95.4	-	-	-	-	-	-
ρ_{u,b_2}	0.25	0.254	0.004	0.002	0.048	0.048	94.6	-	-	-	-	-	-
**Personalized survival probabilities**													
$\Pr(w_k > 6 \text{ months}\|\boldsymbol{w}, \boldsymbol{Y})$	-	-	0.3%	-	-	-	95.4	-	1.1%	-	-	-	23.4
$\Pr(w_k > 12 \text{ months}\|\boldsymbol{w}, \boldsymbol{Y})$	-	-	0.3%	-	-	-	96.2	-	1.4%	-	-	-	24.0
$\Pr(w_k > 18 \text{ months}\|\boldsymbol{w}, \boldsymbol{Y})$	-	-	0.3%	-	-	-	95.4	-	1.3%	-	-	-	23.8
$\Pr(w_k > 24 \text{ months}\|\boldsymbol{w}, \boldsymbol{Y})$	-	-	0.3%	-	-	-	95.4	-	1.2%	-	-	-	23.6

Results from 500 replications with each dataset including 500 individuals. "True" denotes the true parameter values; "Est" the mean of the estimates over the 500 replications; "Bias" the mean bias of estimates; "MSE" the mean squared error; "ASE" the average SE; "MCSD" the empirical Monte Carlo deviation of estimates and "Cov." the empirical coverage probability (%) of the confidence intervals. For the Personalized survival probabilities (where global true values cannot be defined as they depend on specific patients), "Bias" indicates the mean absolute deviation between the true, patient-specific probability and the estimated one, while "Cov." the proportion of simulations in which the estimated confidence interval contained the true, patient-specific probability.

TABLE 8 Simulation study results, where the data have been generated by the proposed model under Scenario II-C. Fitting model (i) is correctly specified ("Correctly specified") and (ii) ignores the longitudinal model ("Ignoring longitudinal model").

**Parameter**	**Correctly Specified**							**Ignoring longitudinal model**					
	**True**	**Est**	**Bias**	**MSE**	**ASE**	**MCSD**	**Cov.**	**Est**	**Bias**	**MSE**	**ASE**	**MCSD**	**Cov.**
**Longitudinal**													
Intercept (β_0)	17.50	17.512	0.012	0.092	0.315	0.303	95.2	-	-	-	-	-	-
t (β_1)	-1.10	-1.096	0.004	0.002	0.050	0.050	94.4	-	-	-	-	-	-
$\sqrt{t}$ (β_2)	6.30	6.286	-0.014	0.038	0.187	0.185	94.8	-	-	-	-	-	-
V_1 (β_3)	1.50	1.502	0.002	0.034	0.187	0.185	94.8	-	-	-	-	-	-
**Visiting Process**													
Observed marker value (γ)	-0.10	-0.100	0.000	0.000	0.003	0.003	95.6	-0.083	0.017	0.000	0.003	0.003	00.0
V_2 (ϕ)	0.50	0.501	0.001	0.003	0.056	0.053	96.2	0.505	0.005	0.021	0.149	0.146	95.6
**Variance components**													
σ_u^2	3	3.001	0.001	0.045	0.209	0.212	94.8	2.693	-0.307	0.038	0.192	0.195	66.2
ρ_{u,b_0}	0.70	0.701	0.001	0.001	0.025	0.024	95.0	-	-	-	-	-	-
ρ_{u,b_1}	-0.20	-0.199	0.001	0.002	0.050	0.049	95.4	-	-	-	-	-	-
ρ_{u,b_2}	0.25	0.248	-0.002	0.002	0.048	0.047	96.4	-	-	-	-	-	-
**Personalized survival probabilities**													
$\Pr(w_k > 6 \text{ months} \mid \boldsymbol{w}, \boldsymbol{Y})$	-	-	0.7%	-	-	-	95.4	-	3.9%	-	-	-	10.2
$\Pr(w_k > 12 \text{ months} \mid \boldsymbol{w}, \boldsymbol{Y})$	-	-	0.7%	-	-	-	95.0	-	4.3%	-	-	-	9.4
$\Pr(w_k > 18 \text{ months} \mid \boldsymbol{w}, \boldsymbol{Y})$	-	-	0.6%	-	-	-	95.4	-	4%	-	-	-	11.4
$\Pr(w_k > 24 \text{ months} \mid \boldsymbol{w}, \boldsymbol{Y})$	-	-	0.5%	-	-	-	95.4	-	3.7%	-	-	-	11.2

Results from 500 replications with each dataset including 500 individuals. “True” denotes the true parameter values; “Est” the mean of the estimates over the 500 replications; “Bias” the mean bias of estimates; “MSE” the mean squared error; “ASE” the average SE; “MCSD” the empirical Monte Carlo deviation of estimates and “Cov.” the empirical coverage probability (%) of the confidence intervals. For the Personalized survival probabilities (where global true values cannot be defined as they depend on specific patients), “Bias” indicates the mean absolute deviation between the true, patient-specific probability and the estimated one, while “Cov.” the proportion of simulations in which the estimated confidence interval contained the true, patient-specific probability.

TABLE 9 Simulation study results, where the data have been generated by the proposed model under Scenario II-B. Fitting model (i) is correctly specified ("Correctly specified") and (ii) ignores the longitudinal model ("Ignoring longitudinal model").

Parameter	Correctly Specified							Ignoring longitudinal model					
	**True**	**Est**	**Bias**	**MSE**	**ASE**	**MCSD**	**Cov.**	**Est**	**Bias**	**MSE**	**ASE**	**MCSD**	**Cov.**
**Longitudinal**													
Intercept (β_0)	17.50	17.499	-0.001	0.148	0.380	0.385	95.0	-	-	-	-	-	-
t (β_1)	-1.10	-1.100	0.000	0.002	0.050	0.049	96.0	-	-	-	-	-	-
$\sqrt{t}$ (β_2)	6.30	6.302	0.002	0.036	0.195	0.189	95.8	-	-	-	-	-	-
V_1 (β_3)	1.50	1.495	-0.005	0.199	0.467	0.446	96.2	-	-	-	-	-	-
**Visiting Process**													
Observed marker value (γ)	-0.10	-0.100	0.000	0.000	0.003	0.003	93.0	-0.093	0.007	0.000	0.003	0.003	42.5
V_2 (ϕ)	0.50	0.503	0.003	0.021	0.139	0.143	94.4	0.510	0.010	0.025	0.155	0.158	94.4
**Variance components**													
σ_u^2	3	2.994	-0.006	0.042	0.204	0.205	94.6	2.938	-0.062	0.047	0.199	0.216	94.0
ρ_{u,b_0}	0.25	0.254	0.004	0.002	0.044	0.042	94.6	-	-	-	-	-	-
ρ_{u,b_1}	-0.20	-0.197	0.003	0.002	0.050	0.049	95.6	-	-	-	-	-	-
ρ_{u,b_2}	0.25	0.248	-0.002	0.002	0.048	0.046	95.6	-	-	-	-	-	-
**Personalized survival probabilities**													
$\Pr(w_k > 6 \text{ months} \mid \boldsymbol{w}, \boldsymbol{Y})$	-	-	0.3%	-	-	-	94.4	-	0.7%	-	-	-	39.9
$\Pr(w_k > 12 \text{ months} \mid \boldsymbol{w}, \boldsymbol{Y})$	-	-	0.3%	-	-	-	95.4	-	0.8%	-	-	-	40.1
$\Pr(w_k > 18 \text{ months} \mid \boldsymbol{w}, \boldsymbol{Y})$	-	-	0.3%	-	-	-	94.4	-	0.8%	-	-	-	38.1
$\Pr(w_k > 24 \text{ months} \mid \boldsymbol{w}, \boldsymbol{Y})$	-	-	0.3%	-	-	-	94.6	-	0.7%	-	-	-	37.3

Results from 500 replications with each dataset including 500 individuals. "True" denotes the true parameter values; "Est" the mean of the estimates over the 500 replications; "Bias" the mean bias of estimates; "MSE" the mean squared error; "ASE" the average SE; "MCSD" the empirical Monte Carlo deviation of estimates and "Cov." the empirical coverage probability (%) of the confidence intervals. For the Personalized survival probabilities (where global true values cannot be defined as they depend on specific patients), "Bias" indicates the mean absolute deviation between the true, patient-specific probability and the estimated one, while "Cov." the proportion of simulations in which the estimated confidence interval contained the true, patient-specific probability.

TABLE 10 Simulation study results, where the data have been generated by the proposed model under Scenario II-D. Fitting model (i) is correctly specified ("Correctly specified") and (ii) ignores the longitudinal model ("Ignoring longitudinal model").

**Parameter**				**Correctly Specified**							**Ignoring longitudinal model**		
	**True**	**Est**	**Bias**	**MSE**	**ASE**	**MCSD**	**Cov.**	**Est**	**Bias**	**MSE**	**ASE**	**MCSD**	**Cov.**
**Longitudinal**													
Intercept (β_0)	17.50	17.516	0.016	0.139	0.372	0.374	95.6	-	-	-	-	-	-
t (β_1)	-1.10	-1.101	-0.001	0.003	0.050	0.051	94.0	-	-	-	-	-	-
$\sqrt{t}$ (β_2)	6.30	6.299	-0.001	0.038	0.194	0.196	94.6	-	-	-	-	-	-
V_1 (β_3)	1.50	1.493	-0.007	0.200	0.439	0.448	94.6	-	-	-	-	-	-
**Visiting Process**													
Observed marker value (γ)	-0.10	-0.100	0.000	0.000	0.003	0.003	93.6	-0.092	0.008	0.000	0.003	0.003	27.8
V_2 (ϕ)	0.50	0.509	0.009	0.015	0.123	0.122	92.8	0.507	0.007	0.025	0.155	0.157	94.0
**Variance components**													
σ_u^2	3	3.001	0.001	0.044	0.205	0.210	93.4	2.918	-0.082	0.042	0.199	0.205	90.8
ρ_{u,b_0}	0.45	0.451	0.001	0.001	0.038	0.037	94.6	-	-	-	-	-	-
ρ_{u,b_1}	-0.40	-0.398	0.002	0.002	0.045	0.042	96.8	-	-	-	-	-	-
ρ_{u,b_2}	0.25	0.248	-0.002	0.002	0.048	0.045	95.2	-	-	-	-	-	-
**Personalized survival probabilities**													
$\Pr(w_k > 6 \text{ months}\vert \boldsymbol{w}, \boldsymbol{Y})$	-	-	0.2%	-	-	-	95.4	-	1%	-	-	-	27.5
$\Pr(w_k > 12 \text{ months}\vert \boldsymbol{w}, \boldsymbol{Y})$	-	-	0.3%	-	-	-	95.4	-	1.2%	-	-	-	26.7
$\Pr(w_k > 18 \text{ months}\vert \boldsymbol{w}, \boldsymbol{Y})$	-	-	0.3%	-	-	-	95.6	-	1.1%	-	-	-	27.9
$\Pr(w_k > 24 \text{ months}\vert \boldsymbol{w}, \boldsymbol{Y})$	-	-	0.3%	-	-	-	95.4	-	1.1%	-	-	-	26.7

Results from 500 replications with each dataset including 500 individuals. "True" denotes the true parameter values; "Est" the mean of the estimates over the 500 replications; "Bias" the mean bias of estimates; "MSE" the mean squared error; "ASE" the average SE; "MCSD" the empirical Monte Carlo deviation of estimates and "Cov." the empirical coverage probability (%) of the confidence intervals. For the Personalized survival probabilities (where global true values cannot be defined as they depend on specific patients), "Bias" indicates the mean absolute deviation between the true, patient-specific probability and the estimated one, while "Cov." the proportion of simulations in which the estimated confidence interval contained the true, patient-specific probability.

TABLE 11 Simulation study results for personalized survival probabilities stratified by the number of previous visits, where the data have been generated by the proposed model under Scenario II-A. Fitting model (i) is correctly specified ("Correctly specified") and (ii) ignores the longitudinal model ("Ignoring longitudinal model").

Parameter	Correctly Specified		Ignoring longitudinal model	
	**Bias**	**Cov.**	**Bias**	**Cov.**
**Personalized survival probabilities**				
$k \leq 12$				
$\Pr(w_k > 6 \text{ months} \mid \boldsymbol{w}, \boldsymbol{Y})$	0.2%	96.8	0.6%	34.0
$\Pr(w_k > 12 \text{ months} \mid \boldsymbol{w}, \boldsymbol{Y})$	0.6%	96.8	2.2%	34.6
$\Pr(w_k > 18 \text{ months} \mid \boldsymbol{w}, \boldsymbol{Y})$	0.8%	96.2	2.8%	31.4
$\Pr(w_k > 24 \text{ months} \mid \boldsymbol{w}, \boldsymbol{Y})$	0.8%	96.8	2.9%	30.1
$12 < k \leq 18$				
$\Pr(w_k > 6 \text{ months} \mid \boldsymbol{w}, \boldsymbol{Y})$	0.2%	97.1	0.9%	22.1
$\Pr(w_k > 12 \text{ months} \mid \boldsymbol{w}, \boldsymbol{Y})$	0.4%	97.1	1.7%	23.5
$\Pr(w_k > 18 \text{ months} \mid \boldsymbol{w}, \boldsymbol{Y})$	0.3%	95.6	1.2%	25.0
$\Pr(w_k > 24 \text{ months} \mid \boldsymbol{w}, \boldsymbol{Y})$	0.2%	95.6	0.8%	24.3
$k > 18$				
$\Pr(w_k > 6 \text{ months} \mid \boldsymbol{w}, \boldsymbol{Y})$	0.4%	93.3	1.6%	16.3
$\Pr(w_k > 12 \text{ months} \mid \boldsymbol{w}, \boldsymbol{Y})$	0.1%	95.2	0.5%	16.3
$\Pr(w_k > 18 \text{ months} \mid \boldsymbol{w}, \boldsymbol{Y})$	0.0%	94.7	0.2%	17.3
$\Pr(w_k > 24 \text{ months} \mid \boldsymbol{w}, \boldsymbol{Y})$	0.0%	94.2	0.1%	18.3

"Bias" indicates the mean absolute deviation between the true, patient-specific probability and the estimated one, while "Cov." the proportion of simulations in which the estimated confidence interval contained the true, patient-specific probability, k denotes the number of previous visits.

TABLE 12 Simulation study results for personalized survival probabilities stratified by the number of previous visits, where the data have been generated by the proposed model under Scenario II-C. Fitting model (i) is correctly specified ("Correctly specified") and (ii) ignores the longitudinal model ("Ignoring longitudinal model").

**Parameter**	**Correctly Specified**		**Ignoring longitudinal model**	
	**Bias**	**Cov.**	**Bias**	**Cov.**
**Personalized survival probabilities**				
$k \leq 12$				
$\Pr(w_k > 6 \text{ months} \mid \boldsymbol{w}, \boldsymbol{Y})$	0.3%	94.8	2.3%	6.5
$\Pr(w_k > 12 \text{ months} \mid \boldsymbol{w}, \boldsymbol{Y})$	1.2%	92.8	8.6%	6.5
$\Pr(w_k > 18 \text{ months} \mid \boldsymbol{w}, \boldsymbol{Y})$	1.4%	93.5	10.4%	8.5
$\Pr(w_k > 24 \text{ months} \mid \boldsymbol{w}, \boldsymbol{Y})$	1.4%	94.1	10.5%	7.8
$12 < k \leq 18$				
$\Pr(w_k > 6 \text{ months} \mid \boldsymbol{w}, \boldsymbol{Y})$	0.4%	96.5	2.0%	19.0
$\Pr(w_k > 12 \text{ months} \mid \boldsymbol{w}, \boldsymbol{Y})$	0.6%	95.8	3.3%	17.6
$\Pr(w_k > 18 \text{ months} \mid \boldsymbol{w}, \boldsymbol{Y})$	0.5%	95.8	2.2%	21.1
$\Pr(w_k > 24 \text{ months} \mid \boldsymbol{w}, \boldsymbol{Y})$	0.3%	95.8	1.3%	17.6
$k > 18$				
$\Pr(w_k > 6 \text{ months} \mid \boldsymbol{w}, \boldsymbol{Y})$	1.1%	95.1	6.5%	6.8
$\Pr(w_k > 12 \text{ months} \mid \boldsymbol{w}, \boldsymbol{Y})$	0.3%	96.1	1.7%	5.9
$\Pr(w_k > 18 \text{ months} \mid \boldsymbol{w}, \boldsymbol{Y})$	0.1%	96.6	0.5%	6.8
$\Pr(w_k > 24 \text{ months} \mid \boldsymbol{w}, \boldsymbol{Y})$	0.0%	96.1	0.2%	9.3

"Bias" indicates the mean absolute deviation between the true, patient-specific probability and the estimated one, while "Cov." the proportion of simulations in which the estimated confidence interval contained the true, patient-specific probability, k denotes the number of previous visits.

TABLE 13 Simulation study results for personalized survival probabilities stratified by the number of previous visits, where the data have been generated by the proposed model under Scenario II-B. Fitting model (i) is correctly specified ("Correctly specified") and (ii) ignores the longitudinal model ("Ignoring longitudinal model").

**Parameter**	**Correctly Specified**		**Ignoring longitudinal model**	
	**Bias**	**Cov.**	**Bias**	**Cov.**
**Personalized survival probabilities**				
$k \leq 12$				
$\Pr(w_k > 6 \text{ months}\mid \boldsymbol{w}, \boldsymbol{Y})$	0.2%	94.3	0.3%	49.7
$\Pr(w_k > 12 \text{ months}\mid \boldsymbol{w}, \boldsymbol{Y})$	0.6%	96.0	1.3%	50.3
$\Pr(w_k > 18 \text{ months}\mid \boldsymbol{w}, \boldsymbol{Y})$	0.7%	94.9	1.7%	49.1
$\Pr(w_k > 24 \text{ months}\mid \boldsymbol{w}, \boldsymbol{Y})$	0.8%	95.4	1.8%	48.0
$12 < k \leq 18$				
$\Pr(w_k > 6 \text{ months}\mid \boldsymbol{w}, \boldsymbol{Y})$	0.2%	91.4	0.7%	37.1
$\Pr(w_k > 12 \text{ months}\mid \boldsymbol{w}, \boldsymbol{Y})$	0.4%	92.2	1.1%	36.2
$\Pr(w_k > 18 \text{ months}\mid \boldsymbol{w}, \boldsymbol{Y})$	0.3%	91.4	0.7%	33.6
$\Pr(w_k > 24 \text{ months}\mid \boldsymbol{w}, \boldsymbol{Y})$	0.2%	91.4	0.4%	32.8
$k > 18$				
$\Pr(w_k > 6 \text{ months}\mid \boldsymbol{w}, \boldsymbol{Y})$	0.5%	96.2	1.1%	33.2
$\Pr(w_k > 12 \text{ months}\mid \boldsymbol{w}, \boldsymbol{Y})$	0.1%	96.7	0.4%	33.7
$\Pr(w_k > 18 \text{ months}\mid \boldsymbol{w}, \boldsymbol{Y})$	0.0%	95.7	0.1%	31.2
$\Pr(w_k > 24 \text{ months}\mid \boldsymbol{w}, \boldsymbol{Y})$	0.0%	95.7	0.0%	30.8

"Bias" indicates the mean absolute deviation between the true, patient-specific probability and the estimated one, while "Cov." the proportion of simulations in which the estimated confidence interval contained the true, patient-specific probability, k denotes the number of previous visits.

TABLE 14 Simulation study results for personalized survival probabilities stratified by the number of previous visits, where the data have been generated by the proposed model under Scenario II-D. Fitting model (i) is correctly specified ("Correctly specified") and (ii) ignores the longitudinal model ("Ignoring longitudinal model").

**Parameter**	**Correctly Specified**		**Ignoring longitudinal model**	
	**Bias**	**Cov.**	**Bias**	**Cov.**
**Personalized survival probabilities**				
$k \leq 12$				
$\Pr(w_k > 6 \text{ months}\mid \boldsymbol{w}, \boldsymbol{Y})$	0.1%	97.2	0.5%	38.1
$\Pr(w_k > 12 \text{ months}\mid \boldsymbol{w}, \boldsymbol{Y})$	0.5%	95.0	1.8%	37.0
$\Pr(w_k > 18 \text{ months}\mid \boldsymbol{w}, \boldsymbol{Y})$	0.7%	96.1	2.3%	37.6
$\Pr(w_k > 24 \text{ months}\mid \boldsymbol{w}, \boldsymbol{Y})$	0.8%	96.1	2.4%	37.6
$12 < k \leq 18$				
$\Pr(w_k > 6 \text{ months}\mid \boldsymbol{w}, \boldsymbol{Y})$	0.2%	97.0	1.0%	17.8
$\Pr(w_k > 12 \text{ months}\mid \boldsymbol{w}, \boldsymbol{Y})$	0.4%	97.0	1.8%	18.8
$\Pr(w_k > 18 \text{ months}\mid \boldsymbol{w}, \boldsymbol{Y})$	0.3%	97.0	1.2%	23.8
$\Pr(w_k > 24 \text{ months}\mid \boldsymbol{w}, \boldsymbol{Y})$	0.2%	97.0	0.8%	23.8
$k > 18$				
$\Pr(w_k > 6 \text{ months}\mid \boldsymbol{w}, \boldsymbol{Y})$	0.4%	93.1	1.4%	23.0
$\Pr(w_k > 12 \text{ months}\mid \boldsymbol{w}, \boldsymbol{Y})$	0.1%	94.9	0.4%	21.7
$\Pr(w_k > 18 \text{ months}\mid \boldsymbol{w}, \boldsymbol{Y})$	0.0%	94.5	0.1%	21.7
$\Pr(w_k > 24 \text{ months}\mid \boldsymbol{w}, \boldsymbol{Y})$	0.0%	94.0	0.0%	18.9

"Bias" indicates the mean absolute deviation between the true, patient-specific probability and the estimated one, while "Cov." the proportion of simulations in which the estimated confidence interval contained the true, patient-specific probability, k denotes the number of previous visits.

2.3 Alternative joint model scenario

TABLE 15 Simulation study results, where the data have been generated a joint model in which visiting process depends on marker's random intercept and rate of change of the underlying marker trajectory. Fitting model (i) is the misspecified proposed joint model ("Proposed Model"),(ii) the misspecified proportional hazards model that totally ignores the longitudinal model ("Ignoring longitudinal model") and (iii) the misspecified linear mixed model that totally ignores the visiting process ("Ignoring visiting process").

Parameter				Proposed Model						Ignoring longitudinal model			
	True	Est	Bias	MSE	ASE	MCSD	Cov.	Est	Bias	MSE	ASE	MCSD	Cov.
**Visiting Process**													
Observed marker value (γ)	-0.10	-0.104	-	-	0.006	-	-	-0.073	-	-	0.005	-	-
V_2 (ϕ)	0.50	0.490	-	-	0.101	-	-	0.488	-	-	0.146	-	-
**Personalized survival probabilities**													
$\Pr(w_k > 6 \text{ months}\|\boldsymbol{w}, \boldsymbol{Y})$	-	-	2%	-	-	-	12.4	-	2.4%	-	-	-	8.7
$\Pr(w_k > 12 \text{ months}\|\boldsymbol{w}, \boldsymbol{Y})$	-	-	2.9%	-	-	-	26.7	-	3.6%	-	-	-	15.4
$\Pr(w_k > 18 \text{ months}\|\boldsymbol{w}, \boldsymbol{Y})$	-	-	2.3%	-	-	-	41.4	-	2.9%	-	-	-	21.4
$\Pr(w_k > 24 \text{ months}\|\boldsymbol{w}, \boldsymbol{Y})$	-	-	2.2%	-	-	-	41.1	-	2.9%	-	-	-	21.2
				**Proposed Model**						**Ignoring visiting process**			
**Longitudinal**													
Intercept (β_0)	17.50	17.545	0.045	0.204	0.449	0.453	95.2	18.30	-0.80	0.264	0.480	0.515	58.0
t (β_1)	-1.10	-1.098	0.002	0.004	0.069	0.067	96.1	-0.956	0.144	0.005	0.066	0.067	43.0
$\sqrt{t}$ (β_2)	6.30	6.295	-0.005	0.079	0.295	0.282	95.9	5.662	-0.638	0.081	0.280	0.285	39.2
V_1 (β_3)	1.50	1.456	-0.044	0.138	0.367	0.372	94.7	1.461	-0.039	0.335	0.539	0.579	93.5

Results from 500 replications with each dataset including 500 individuals. "True" denotes the true parameter values; "Est" the mean of the estimates over the 500 replications; "Bias" the mean bias of estimates; "MSE" the mean squared error; "ASE" the average SE; "MCSD" the empirical Monte Carlo deviation of estimates and "Cov." the empirical coverage probability (%) of the confidence intervals. For the Personalized survival probabilities (where global true values cannot be defined as they depend on specific patients), "Bias" indicates the mean absolute deviation between the true, patient-specific probability and the estimated one, while "Cov." the proportion of simulations in which the estimated confidence interval contained the true, patient-specific probability.

2.4 | Terminal event scenario

TABLE 16 Simulation study results under Scenario II-A with an informative terminal event, targeting approximately 5% terminal events within the study duration. The proposed model was fitted ignoring the terminal event process.

Parameter	Proposed model						
	**True**	**Est**	**Bias**	**MSE**	**ASE**	**MCSD**	**Cov.**
**Longitudinal**							
Intercept (β_0)	17.50	17.508	0.008	0.112	0.358	0.334	97.0
t (β_1)	-1.10	-1.101	-0.001	0.003	0.051	0.052	95.6
$\sqrt{t}$ (β_2)	6.30	6.313	0.013	0.040	0.196	0.201	95.4
V_1 (β_3)	1.50	1.505	0.005	0.160	0.394	0.400	95.4
Mean marker level at $t = 12$ years ($V_1 = 0$)	26.12	26.169	0.045	0.130	0.365	0.357	95.0
Mean marker level at $t = 12$ years ($V_1 = 1$)	27.62	27.674	0.050	0.144	0.364	0.376	93.8
**Visiting Process**							
Observed marker value (γ)	-0.10	-0.100	0.000	0.000	0.003	0.003	95.4
V_2 (ϕ)	0.50	0.492	-0.008	0.014	0.118	0.118	94.8
**Variance components**							
σ_u^2	3.00	2.989	-0.011	0.043	0.207	0.206	94.4
ρ_{u,b_0}	0.45	0.449	-0.001	0.001	0.039	0.038	94.6
ρ_{u,b_1}	-0.20	-0.202	-0.002	0.002	0.051	0.050	95.8
ρ_{u,b_2}	0.25	0.249	-0.001	0.002	0.049	0.049	95.2

Results from 500 replications with each dataset including 500 individuals. "True" denotes the true parameter values; "Est" the mean of the estimates over the 500 replications; "Bias" the mean bias of estimates; "MSE" the mean squared error; "ASE" the average SE; "MCSD" the empirical Monte Carlo deviation of estimates and "Cov." the empirical coverage probability (%) of the confidence intervals.

TABLE 17 Simulation study results under Scenario II-A with an informative terminal event, targeting approximately 40% terminal events within the study duration. The proposed model was fitted ignoring the terminal event process.

**Parameter**	**Proposed model**						
	**True**	**Est**	**Bias**	**MSE**	**ASE**	**MCSD**	**Cov.**
**Longitudinal**							
Intercept (β_0)	17.50	17.517	0.017	0.112	0.362	0.334	96.6
t (β_1)	-1.10	-1.118	-0.018	0.004	0.059	0.060	94.0
$\sqrt{t}$ (β_2)	6.30	6.471	0.171	0.076	0.213	0.217	87.2
V_1 (β_3)	1.50	1.492	-0.008	0.166	0.407	0.408	95.8
Mean marker level at $t = 12$ years ($V_1 = 0$)	26.12	26.516	0.392	0.321	0.401	0.409	79.4
Mean marker level at $t = 12$ years ($V_1 = 1$)	27.62	28.008	0.384	0.333	0.396	0.431	82.8
**Visiting Process**							
Observed marker value (γ)	-0.10	-0.100	0.000	0.000	0.004	0.004	96.4
V_2 (ϕ)	0.50	0.493	-0.007	0.016	0.126	0.126	96.2
**Variance components**							
σ_u^2	3.00	2.907	-0.093	0.058	0.222	0.222	92.2
ρ_{u,b_0}	0.45	0.438	-0.012	0.002	0.043	0.043	94.0
ρ_{u,b_1}	-0.20	-0.191	0.009	0.003	0.059	0.057	95.4
ρ_{u,b_2}	0.25	0.226	-0.024	0.003	0.054	0.054	93.2

Results from 500 replications with each dataset including 500 individuals. "True" denotes the true parameter values; "Est" the mean of the estimates over the 500 replications; "Bias" the mean bias of estimates; "MSE" the mean squared error; "ASE" the average SE; "MCSD" the empirical Monte Carlo deviation of estimates and "Cov." the empirical coverage probability (%) of the confidence intervals.

2.5 | Binary longitudinal marker scenario

TABLE 18 Simulation study results, where the data have been generated by the proposed model and the longitudinal marker is binary. Fitting model is correctly specified ("Correctly specified").

**Parameter**	**Correctly Specified**						
	**True**	**Est**	**Bias**	**MSE**	**ASE**	**MCSD**	**Cov.**
**Longitudinal**							
Intercept (β_0)	-2	-2.039	-0.039	0.634	0.786	0.797	94.5
t (β_1)	-0.3	-0.301	-0.001	0.000	0.011	0.012	92.9
V_1 (β_2)	0.1	0.102	0.002	0.001	0.026	0.026	94.7
**Visiting Process**							
Observed marker value (γ)	-4	-4.002	-0.002	0.005	0.068	0.073	93.3
V_2 (ϕ)	0.50	0.499	-0.001	0.022	0.145	0.149	94.5
**Variance components**							
σ_u^2	3	2.984	-0.016	0.041	0.212	0.204	94.7
ρ_{u,b_0}	-0.5	-0.497	0.003	0.002	0.045	0.045	95.5

Results from 500 replications with each dataset including 500 individuals. “True” denotes the true parameter values; “Est” the mean of the estimates over the 500 replications; “Bias” the mean bias of estimates; “MSE” the mean squared error; “ASE” the average SE; “MCSD” the empirical Monte Carlo deviation of estimates and “Cov.” the empirical coverage probability (%) of the confidence intervals. In this scenario, $V_1 \sim N(30, 4)$.

3 | WEB APPENDIX C: MODEL PERFORMANCE IN THE VALIDATION SAMPLE OF AMACS DATA

TABLE 19 AUC and prediction error (PE) for the proposed joint model (JM) and a simpler proportional hazards model for the visiting process (VM) in the validation sample of AMACS data .

**AUC**		**PE**	
**JM**	**VM**	**JM**	**VM**
$\Delta_t = 9$ **months**			
0.643	0.635	0.226	0.229
$\Delta_t = 12$ **months**			
0.632	0.605	0.161	0.162

For each individual, the probability of their last uncensored gap time exceeding Δ_t, $\mathbb{P}(w_k > \Delta_t \mid \boldsymbol{w}, \boldsymbol{Y})$, was calculated using all previously observed gap times ($\boldsymbol{w}$) and longitudinal marker values ($\boldsymbol{Y}$). Prediction error was calculated as the average of the squared differences between the predicted probabilities and the true status (whether the next gap-time exceeded Δ_t or not). For varying thresholds of the predicted probabilities, sensitivity and specificity were calculated, and area under the ROC curve (AUC) was obtained.
